# Comparative Study of Potentially Toxic Nickel and Their Potential Human Health Risks in Seafood (Fish and Mollusks) from Peninsular Malaysia

**DOI:** 10.3390/biology11030376

**Published:** 2022-02-27

**Authors:** Chee Kong Yap, Khalid Awadh Al-Mutairi

**Affiliations:** 1 Department of Biology, Faculty of Science, University Putra Malaysia, 43400 UPM Serdang, Malaysia; 2 Department of Biology, Faculty of Science, University of Tabuk, Tabuk 741, Saudi Arabia; kmutairi@ut.edu.sa

**Keywords:** commercial seafood, health risks, fish and mollusks, toxic nickel

## Abstract

**Simple Summary:**

Exposure to highly nickel (Ni)-polluted environments through oral ingestion of seafood may induce a variety of pathological and toxicological impacts, which is our main public concern. The present biomonitoring study concluded that the Ni levels of the three seafood types were found to have posed no Ni’s non-carcinogenic risk of seafood ingestion. In addition, both the average and high-level consumptions of seafood would not pose adverse effects of Ni to the consumers. This study provided a scientific basis for the food safety assessment of Ni and suggestions for risk management of potentially toxic Ni of seafood consumption in Malaysia.

**Abstract:**

Human exposure to highly nickel (Ni)-polluted environments through oral ingestion pathways may cause various pathological effects. This biomonitoring study aimed to assess the human health risk of potentially toxic Ni in 19 species of marine fishes from Setiu (Terengganu) and two popular seafood molluscs (mangrove snail *Cerithidea obtusa* and cockle *Anadara granosa*) from the coastal area of Peninsular Malaysia. The Ni levels of the three seafood types were found below the maximum permissible limit for Ni. The Ni target hazard quotient values of all seafood were lower than 1.00 for average and high-level (AHL) Malaysian consumers, indicating no Ni’s non-carcinogenic risk of seafood consumption. It was also found that the calculated values of estimated weekly intake were below than established provisional tolerable weekly intake of Ni for both AHL consumers. It can be concluded that both the AHL consumption of seafood would not pose adverse effects of Ni to the consumers. This study provided a scientific basis for the food safety assessment of Ni and suggestions for risk management of potentially toxic Ni of seafood consumption in Malaysia.

## 1. Introduction

The 28th element in the periodic table, nickel (Ni), is a transition metal found widely in the environment, including air, water, and soil. It can exist in a variety of oxidation levels (ranging from −1 to +4), although the +2 oxidation state (Ni^2+^) is the most common in the environment and biological systems [1,2,3]. Even though there is no proof that Ni has a physiological purpose or nutritional benefit in humans [4], it has been recognized as a vital and significant element in several microbes, plants, and animal species [5,6,7,8]. Both Ni and Ni compounds are widely utilized in stainless steel, alloys, rubber and plastic industries, Ni-cadmium battery industries, and electroplating industries [3,9,10,11]. However, the production and use of Ni and its derivatives can expose humans and the environment to different risks with its secondary products polluting the environment at all phases of manufacturing, recycling, and disposal due to the wide distribution of items containing this metal [11].

Human exposure to severely Ni-polluted surroundings may induce a variety of pathological and toxicological impacts, which is our main public concern [12,13,14] since they have been linked to health problems in people who are working with them [15]. Ni is one of the trace elements in Europe that has been listed on the European Commission List-II (Dangerous Substances Directive) and regulated through the Council of European Communities because of its toxicity, persistence, and affinity for bioaccumulation [16]. Oral intake, or ingestion by food, is the most common way for individuals to be exposed to Ni, with an average intake of 100 to 300 µg/day for adults [3,17]. Chronic exposure to Ni and Ni compounds in the body has been linked to adverse negative health effects in humans, including lung fibrosis, renal disease, cardiovascular disease, and respiratory tract cancer [18,19]. Soluble and insoluble Ni compounds are categorized as Group 1 (carcinogenic to people). In contrast, Ni and alloys are classed as Group 2B (probably carcinogenic to humans) by the International Agency for Research on Cancer (IARC) [20]. Hertel et al. [6] present a complete Ni environmental health criteria. The threshold model for Ni carcinogenicity is indirect genotoxic and epigenetic processes [11]. Ni-induced genotoxicity, carcinogenicity, immunotoxicity, and toxicity in metabolically active tissues were discussed by Das et al. [10]. Genchi et al. [4] have published a study of the chemical properties of Ni in humans and the mechanisms of Ni toxicity. All the above information clearly shows the importance and significance of Ni from environmental to human health.

Seafood consumption may be a significant pathway to food contaminants such as heavy metals, pesticides, and phycotoxins [21]. In particular, mollusks such as cockles are filter feeders, making them a significant potential source of human exposure to the potential toxic metals (PTM) [22]. Consequently, human health assessment of shellfish consumption is a fundamental component of seafood safety risk assessments. Such assessments involve accurately estimating shellfish consumption and the elevated concentrations of the PTM of interest [23], which may contradict the multiple health benefits provided by fish and shellfish consumption [24,25,26,27,28,29]. Ni is found in many aquatic foods [30] that binds to protein and nucleic acid in fish and shellfish and may cause toxicity by interfering with Fe metabolism [31]. 

Ni is substantially accumulated in marine mussels, according to Millward et al. [32]. Studies of Ni monitoring have concentrated on sediments [33], mussels *Perna viridis* [34,35], and snails *Telescopium telescopium* over the previous decade [36]. Some studies had been reported in the Ni levels of edible mangrove snail *Cerithidea obtusa,* such as from Can Gio mangrove Biosphere Reserve in Vietnam [37], Peninsular Malaysia [38,39], and Indonesia [40]. For cockle *Anadara granosa*, the Ni levels had been reported from India [41], Thailand coastal waters [42], and intertidal mudflats of Peninsular Malaysia [43,44,45]. However, none of the above studies investigated the target hazard quotient (THQ) for Ni ingestion and estimated weekly intake (EWI) of Ni.

In Malaysia, several monitoring studies of PTMs in the marine fishes have been reported in the literature [46,47,48,49,50,51,52,53,54,55,56,57,58]. However, information on the THQ and EWI of Ni using similar consumption rates and bodyweight of consumers in commercial fish and shellfish on the coastal waters of Peninsular Malaysia is still lacking. The objective of this study was to assess the human health risks of potentially toxic Ni in 19 species of marine fishes from Setiu (Terengganu) and two popular seafood molluscs (cockles *A. granosa* and mangrove snails *C. obtusa*) from Peninsular Malaysia. 

## 2. Materials and Methods

### 2.1. Sample Collection

**Figure 1:**
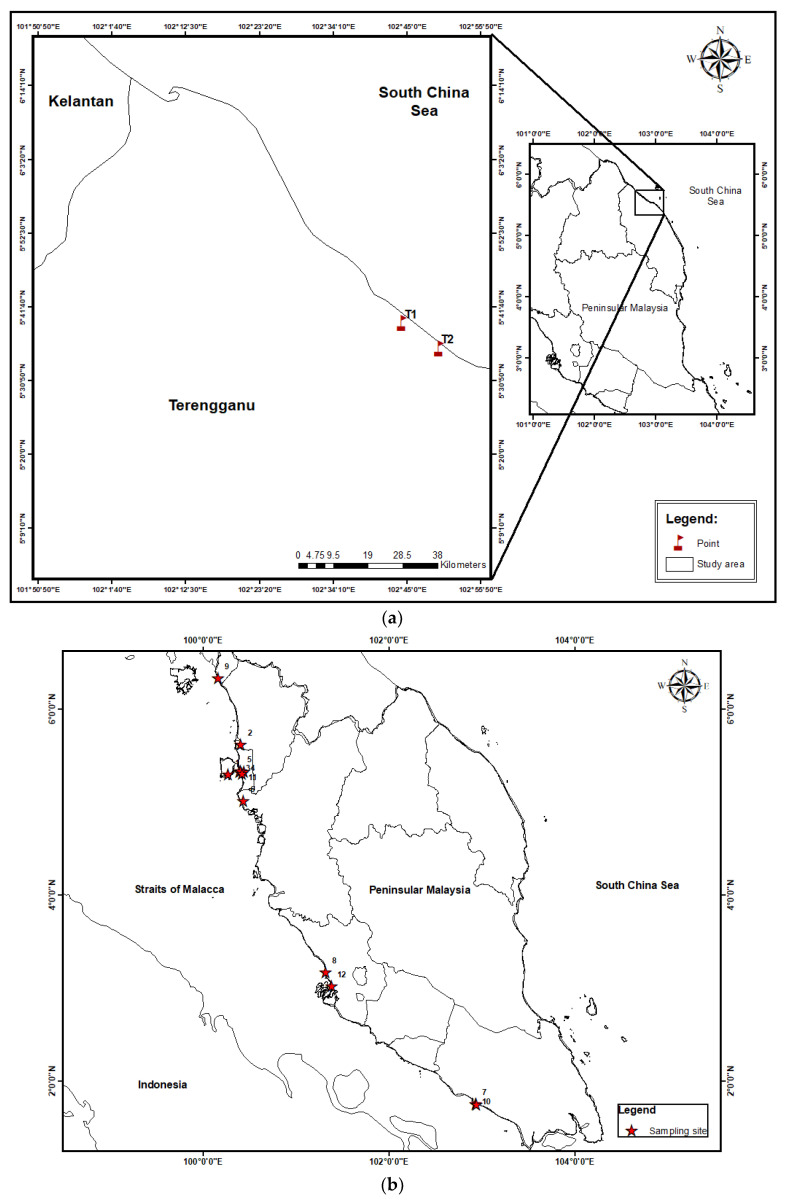

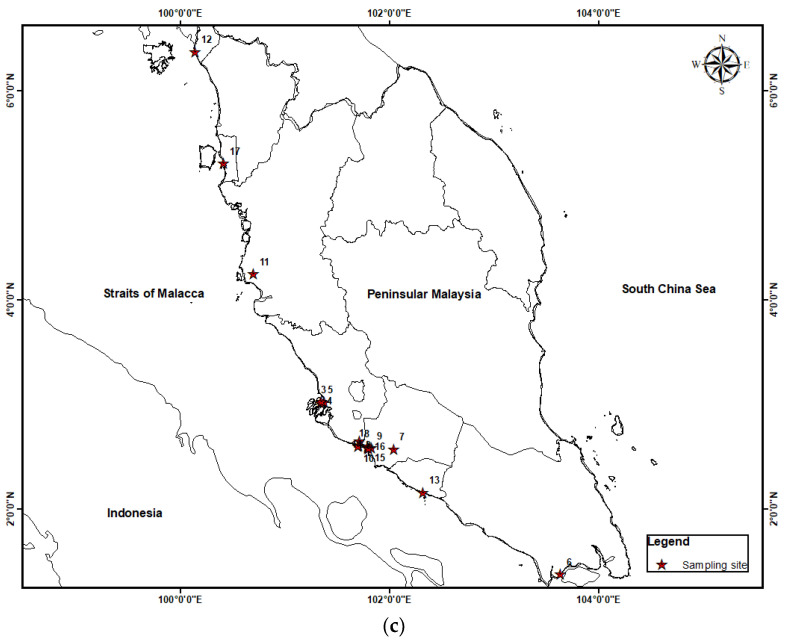
(**a**) Map of fishing loading sites at Kampong Fikri (1) and Kampung Rhu Sepuluh (2) in Setiu, Terengganu, Peninsular Malaysia. (**b**) Sampling map of *Anadara granosa* from 12 sampling sites from the intertidal waters of the west coast of Peninsular Malaysia. (**c**) Sampling map of *Cerithidea obtusa* from 17 sampling sites from the mangrove area of the west coast of Peninsular Malaysia.

Nineteen species of commercial marine fishes were collected from two fishing loading sites: Kampong Fikri (5°39′19″ N, 102°44′16″ E) and Kampung Rhu Sepuluh (5°35′36″ N, 102°49′42″ E) in Setiu (Terengganu, Peninsular Malaysia) (Table S1; Figure 1a). The estimated distance between the sites was about 10 km. The fish were collected directly from fishermen between August 2016 and February 2017. All the fish samples were dead during collection.

The fishes were collected at the landing site and they were assumed to be caught in the vicinity of the east coastal waters of Peninsular Malaysia. For all species, the fishes with similar lengths and weights were collected. The fishes were classified based on information obtained from www.fishbase.org (assessed on 1 August 2017) and key identifications by Mohsin and Ambak [59]. The identification of fish samples was cross-checked based on the online data (https://www.fishbase.in/search.php, assessed on 18 December 2021)) to ensure the species name, family, and habitat niche. 

The cockles A. granosa were collected from 12 sampling sites from the intertidal waters of the west coast of Peninsular Malaysia, between 2005–2008 (Table S2; Figure 1b), while the mangrove snails Cerithidea obtusa were collected from 17 sampling sites from the mangrove area of the west coast of Peninsular Malaysia (between 2006–2010) (Table S3; Figure 1c). 

For comparison purposes, the Ni data in the total soft tissue of green-lipped mussel *P. viridis* from 40 sampling population sites (2002–2009) from the coastal waters of Peninsular Malaysia are cited in Yap et al. [35].

### 2.2. Sample Preparation

Each fish was weighed immediately after collection using a digital electronic balance for fish samples, and the length was measured using a ruler. The length of the fish was measured from the snout on the upper jaw to the end of the tail. Based on a total of 57 individuals (3 replicates of each species) of 19 species of fishes from Setiu, the lengths ranged from 12.5 to 46.0 cm, and the weights ranged from 30 to 335 g. Then, the fishes were dissected for their dorsal muscles. For each fish, 10–20 g dorsal muscle was removed. 

For snail and cockle samples, 20 individuals of almost similar shell lengths (snails: 32.7–49.3 mm; cockles: 23.6–33.9 mm) were selected from each population. The total soft tissues of the mollusks were removed from the shell, and they were pooled from 20 individuals for one population. 

The samples were dried in a lab oven for 72 h at 60 °C until they reached a consistent weight. The samples were then homogenized by grinding them using an agate pestle and mortar. The sample powder was kept in an airtight plastic bag until further investigation.

### 2.3. Metal Analysis

In the digestion tube, 0.50 g of homogenized sample was weighed correctly, followed by 5.0 mL of concentrated nitric acid (HNO_3_; AnalaR grade, BDH 69 percent). They were then cooked for an hour at 40 °C in a hot block digester. After that, the temperature was raised to 140 °C for three hours [35,60]. After digestion, they were diluted with double distilled water before being filtered through filter paper into acid-washed pillboxes (Whatman no 1). An air-acetylene Flame Atomic Absorption Spectrophotometer (FAAS) (Thermo Fisher Scientific- ICE 3000 series AA; USA) was used to determine the Ni concentration in the digested samples. The detection limit of the FAAS for Ni was 0.010 mg/L.

Before usage, all glassware and plastics were soaked overnight in 10% nitric acid, rinsed with distilled water, and dried. Procedure blanks and triplicates of samples were also tested for quality control. The procedure’s correctness was checked using certified reference materials of dogfish liver (DOLT-3, National Research Council Canada). The acquired results were consistent with verified values, demonstrating the method’s repeatability. The recoveries of the CRM obtained were satisfactory (88.8–119%).

### 2.4. Human Health Risk Assessments

For human health risk assessment (HHRA), the Ni data on a dry weight (DW) basis were converted into wet weight (WW) ones using a different fish species conversion factor, as shown in Table 1, while conversion factors of 0.24 and 0.20 were used to convert the DW into WW basis for C. obtusa and A. granosa, respectively. To estimate the HHRA derived from ingesting the fishes and mollusks, four assessments were made, namely:

(a) Direct comparisons with seafood safety guidelines

The comparison was based on only Ni maximum permissible limits (MPL) known as the action level (80 mg/kg WW) for molluscan shellfish (FDA Guidance Document) [61].

(b) Target hazard quotient

To calculate the THQ, the estimated daily intake (EDI) needs to be calculated first. EDI estimates the particular metal intake by using the body weight (bw) and fish consumption rate. It was calculated as in Equation (1):

EDI = (Mc × CR)/bw(1)

where, Mc = metal concentration in the samples (mg/kg) on WW basis; CR = the average-level consumption rates of fish and mollusks (snails, cockles, and mussels) used were 100 and 40 g/person/day, respectively, for Malaysian adults based on 2675 respondents (Malay: 76.9%; Chinese: 14.7%; India: 8.4%) [62]. The high-level consumer rate is assumed to be two times of the average-level consumption rate (as above); bw = body weight of 62 kg for the adult Malaysian population, according to Nurul Izzah et al. [62]. 

Later, the THQ was calculated in Equation (2):

THQ = EDI/ORD(2)

where, ORD = oral reference dose

The oral reference dose (ORD) calculates a contaminant’s daily intake over a lifetime that is unlikely to induce harmful health consequences [63]. In this investigation, the ORD values for Ni = 20 µg/kg/day specified by the US EPA regional screening level [64] were employed.

(c) Comparisons between estimated weekly intake (EWI) and provisional tolerable weekly intake (PTWI)

The Joint FAO/WHO Expert Committee on Food Additives created the provisional tolerated weekly intake (PTWI) [65]. Calculating weekly metal exposures and comparing the results to the relevant prescribed PTWI levels was used to assess the danger to human health posed by food intake. The PTWI is defined as the estimated quantity of a substance in food or drinking water that can be consumed weekly throughout a lifetime without posing a significant health risk, expressed in milligrams per kilogram (mg/kg) of bw [66]. As a result, calculations were carried out to determine how much seafood from this study exceeded the PTWI restrictions. There has been no established PTWI for Ni, but the EFSA [67] recommends a tolerable daily intake (TDI) of 13 µg/kg bw/day [67]. Therefore, the present study set the PTWI for Ni at 91 µg/kg bw/week. Thus, the Ni PTWI for a 62 kg bw for an average adult in Malaysia is equivalent to 5642 µg/week. To estimate the risk of exposure from consuming seafood, the Ni EWI of seafood was calculated as follows:

EWI = EDI × 7

where, EDI = estimated daily intake calculated in Equation (1) and multiplied by seven because there are 7 days in a week.

Comparing calculated EWI and established PTWI limits for a 62 kg adult will determine whether the calculated EWI is below the established Ni PTWI of 5642 µg/week (91 µg/kg bw/week × 62 kg bw). 

In addition, the amount of fish and molluscs that would need to be consumed per week by a 62 kg adult to reach the provisional tolerable weekly intake (PTWI) that was re-calculated based on EFSA [67] was also estimated in the present study. Previously, Yap et al. [35] and Jovic and Stankovic [68] used two levels of mussel consumption values, namely 125 g/week (one meal of mussels every week for average-level mussel consumers or 17.86 g/day) and 250 g/week (one meal of mussels every week for high-level mussel consumers or 35.71 g/day). Hence, based on this understanding, we calculated the fish consumption as 700 g/week (one meal of fish every week for average-level fish consumers or 100 g/day) and 1400 g/week (one meal of fish every week for high-level fish consumers or 200 g/day). For molluscs, we calculated the consumption of snails, cockles and mussels as 280 g/week (one meal of molluscs every week for average-level molluscs consumers or 40 g/day) and 560 g/week (one meal of fish every week for high-level fish consumers or 80 g/day). Subsequently, the values of weekly intake (MWI) of Ni for average and high-level consumers for the fish and molluscs and their percentages to re-calculated PTWI value of Ni were also estimated.

## 3. Results and Discussion 

### 3.1. Comparison with Food Safety Guidelines of Nickel and Reported Studies

**Figure 2:**
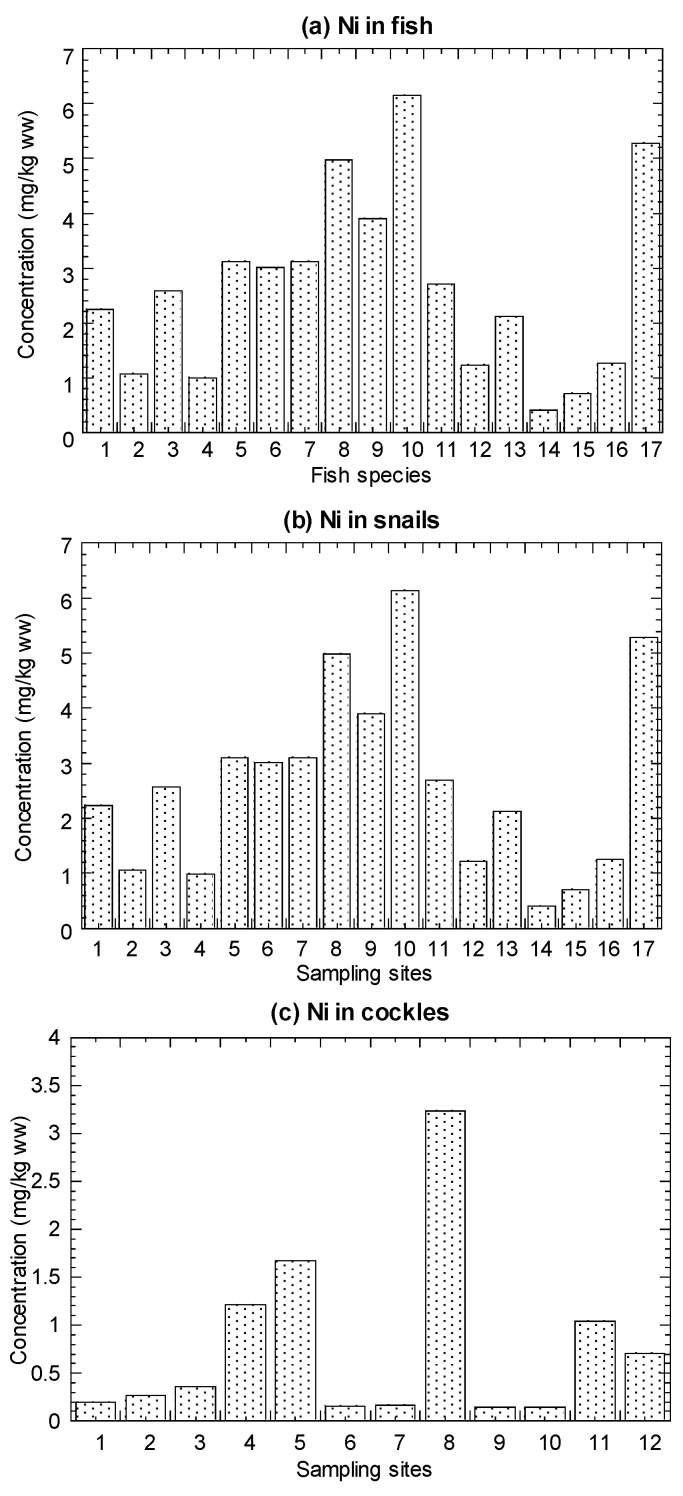

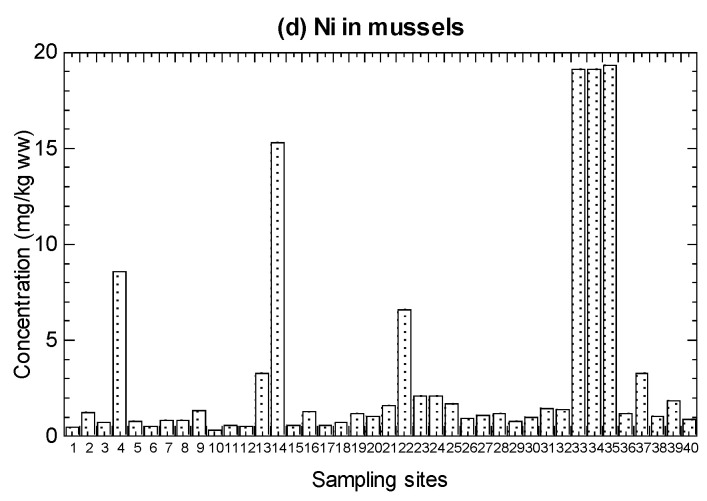
Mean concentrations (mg/kg wet weight (ww)) of nickel (Ni) in the (**a**) marine fish (1–19), (**b**) mangrove snail *Cerithidea obtusa* (1–17), (**c**) cockle *Anadara granosa* (1–12), and (**d**) marine mussel *Perna viridis* (1–40) cited from Yap et al. [35]. Note: The nickel maximum permissible limit = 80 mg/kg ww for molluscan shellfish (FDA Guidance Document) [61].

The values of Ni concentrations in the marine fish, mangrove snails *C. obtusa*, and cockle *A. granosa*, are presented in Figure 2 and summarized in Table 1 (details in Tables S4–S6). 

For the 19 species from Setiu, the Ni concentrations ranged from 0.11–0.90 mg/kg WW (0.48–3.84 mg/kg DW). For *C. obtusa*, the 17 snail populations showed the Ni range between 0.40–6.14 mg/kg WW (1.67–25.6 mg/kg DW), and 12 populations of *A. granosa* ranged from 0.15–3.23 mg/kg WW (0.74–16.2 mg/kg DW) (Table 1). For comparative purpose, the cited Ni in the mussel *P. viridis* from the 40 populations ranged from 0.33–19.3 mg/kg WW (1.94–114 mg/kg DW) (Table S7 and Table 1). 

The literature lacks MPLs for Ni, although the USFDA/CFSAN established the only Ni MPL known as the action level (80 mg/kg WW) for molluscan shellfish (FDA Guidance Document) [61]. As a result, Ni levels in all fish and mollusk species were substantially below the MPL.

The highest and lowest concentrations (mg/kg DW) of Ni were found in *A. maculatus* (3.84) and *M. cordyla* (0.48), respectively (Figure 2). Based on Figure 2, the Ni concentration was highest in *A. maculatus* (3.84), followed by *T. lepturus*, *A. indica*, *N. hexodon*, *A. chacunda*, and others. 

For the fish samples, the comparison of mean Ni concentrations between the present study and reported studies (eight species) of marine fishes in the literature are shown in Table S8. For *Decapterus macrosoma*, the Ni concentrations (mg/kg WW) from the Setiu fish (0.13) were lower than the Gulf of Aqaba (Red Sea) (0.22; [69]). For *Megalaspis cordyla*, the Setiu sample (0.11) was lower than that (0.25) collected from Karachi Fish Harbor in Pakistan [70]. 

For *Otolithes ruber,* the Setiu sample (0.28) was higher than that (0.03) of Chabahar Bay, Makoran (Iran) [27], but lower than those of Kharg Island, Persian Gulf (0.34; [71]), the northern part of the Hormuz strait (Persian Gulf) (2.99; [72]), North of Persian Gulf (0.76; [73]), and Khuzestan shore, northwest of the Persian Gulf (10.1; [74]).

For *Johnius belangerii*, the Setiu sample (0.26) was higher than those of Daya Bay’s Fishery Resource Reserve, South China Sea (0.14; [75]), and Indonesia (0.01; [76]), but lower than those of Musa estuary (0.45 by Monikh et al. [77]; 1.49 by Ravanbakhsh et al. [78]).

For *Pampus chinensis*, the Setiu sample (0.32) was higher than that (0.05) of Cox’s Bazar, Bangladesh [79]. For *Anodontostama chacunda*, the Setiu sample (0.44) was higher than that (0.33) of the Arabian Sea coasts of Pakistan [80]. For *Rastrelliger kanagurta*, the Setiu’ sample (0.17) was higher than that (0.04) of the Kunduchi fish market in Dar es Salaam, Tanzania [81] but lower than that (2.83) of the coastal waters off Kochi, India [82]. For *Scomberomorus commerson,* the Setiu sample (0.35) was higher than that (0.14) of the Coast of Karachi, Pakistan [70], but lower than that (32.2) of Zhongsha (Macclesfield) Fishing Ground, South China Sea [83]. However, the other nine species of fish investigated could not compare with any reported studies because the Ni levels were limited, or none were found in the literature. 

In general, the observed values of Ni (0.48–3.84 mg/kg DW) in 19 species of fish muscles from Setiu were lower than those based on eight species of marine species (among the 19 species in Setiu samples) (Table S9). The present Nil range in fish was also lower than the values reported from the Mediterranean Sea (4.25–6.07 mg/kg DW) [84]; southwest coast of India (6.06–13.92 mg/kg DW) [82], Iran (49.40–54.10 mg/kg DW) [74], and seven marine fishes from Nigeria as 1.57–4.36 mg/kg DW [85]. 

For *C. obtusa*, the present Ni range (1.67–25.6 mg/kg DW) (0.40–6.14 mg/kg WW) was higher than that (5.93–6.15 mg/kg DW) collected from Can Gio mangrove Biosphere Reserve in Vietnam [37], Pontian, of Peninsular Malaysia (1.20 mg/kg DW; [39]), and Singkep Island of Indonesia (9.99–13.1 mg/kg DW; [40]). Yap and Edward [38] reported the Ni levels in the different parts of *C. obtusa* and did not report the total soft tissue. Therefore, the Ni level in the present study analyzed in the total soft tissues is incomparable to those reported by Yap and Edward [38]. 

For *A. granosa*, the present range of Ni levels (0.74–16.2 mg/kg DW) was higher and within those of *A. granosa* reported from Bombay Harbour (1976–1980) (3.90–10.8 mg/kg DW) [41], Thailand coastal waters (1.3–2.00 mg/kg DW) [42], and intertidal mudflats of Peninsular Malaysia (0.74–1.35 mg/kg DW) [43], but lower based on three cockle farms (Kuala Juru, Kuala Sepetang, and dan Kuala Selangor) in Peninsular Malaysia (8.3–20.8 mg/kg DW; [45]). The Ni range (0.15–3.23 mg/kg WW) was also higher than that reported from the retail outlets in Kuala Lumpur (Malaysia) (0.29–0.54 mg/kg WW) [44] and Pantai Jeram cockle farm (0.06–0.18 mg/kg WW; [86]). 

### 3.2. Comparisons of Nickel Target Hazard Quotients among Fish, Snail, Cockle, and Mussel

The Ni data cited from the literature were recalculated for EDI and THQ based on fish consumption rate (100 g/person/day) and bw of 62 kg for the adult Malaysian population, according to Nurul Izzah et al. [62]. The data initially reported on a DW basis were all converted into a WW basis based on the conversion factor for each species from this study. 

**Figure 3:**
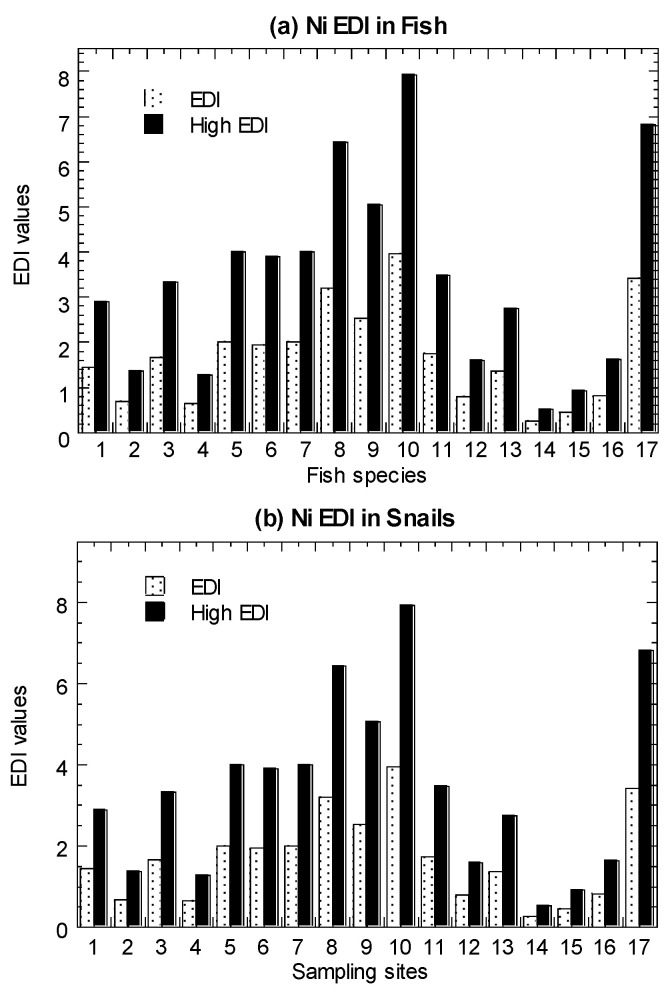

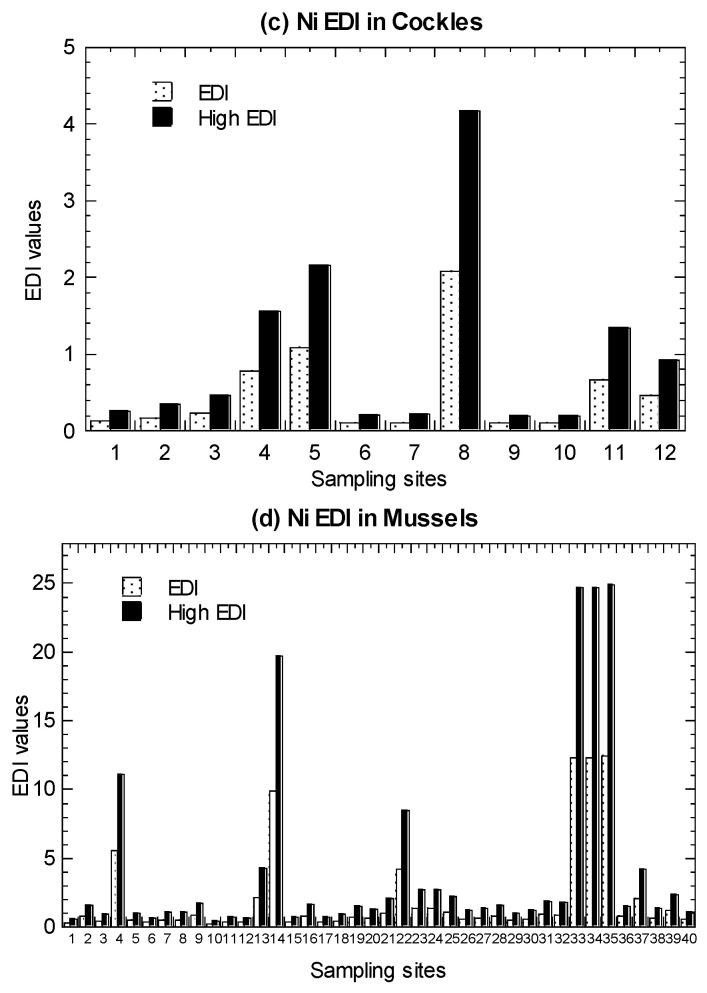
Values of average-level estimated daily intake (EDI) and high-level estimated daily intake (High EDI) of nickel (Ni) in the (**a**) marine fish (1–19), (**b**) mangrove snail *Cerithidea obtusa* (1–17), (**c**) cockle *Anadara granosa* (1–12), and (**d**) marine mussel *Perna viridis* (1–40) cited from Yap et al. [35]. Note: The nickel oral reference dose = 20 µg/kg/day [64]. The high-level consumers (2 times the consumption rate of average-level consumers) are indicated by High.

Values of average-level EDI and high-level EDI in the marine fish, mangrove snails, cockles, and mussels are presented in Figure 3 and summarized in Table 1 (Tables S4–S7). In both the average and high-level consumers, for fish, they were found to have EDI values ranging from 0.18–2.90. Based on eight marine species (among the 19 species in Setiu samples) (Tables S9 and S10), the EDI values for average-level consumers ranged from 0.01–52.1.

For *C. obtusa*, the EDI values ranged from 0.26–7.92. For *A. granosa*, the EDI values ranged from 0.10–4.17. For the cited Ni data for *P. viridis*, the EDI values ranged from 0.21–24.95. These values were lower than Ni ORD (20.0 µg/kg/day), except for high-level consumers of the mussel samples collected from the polluted sites [35].

**Figure 4:**
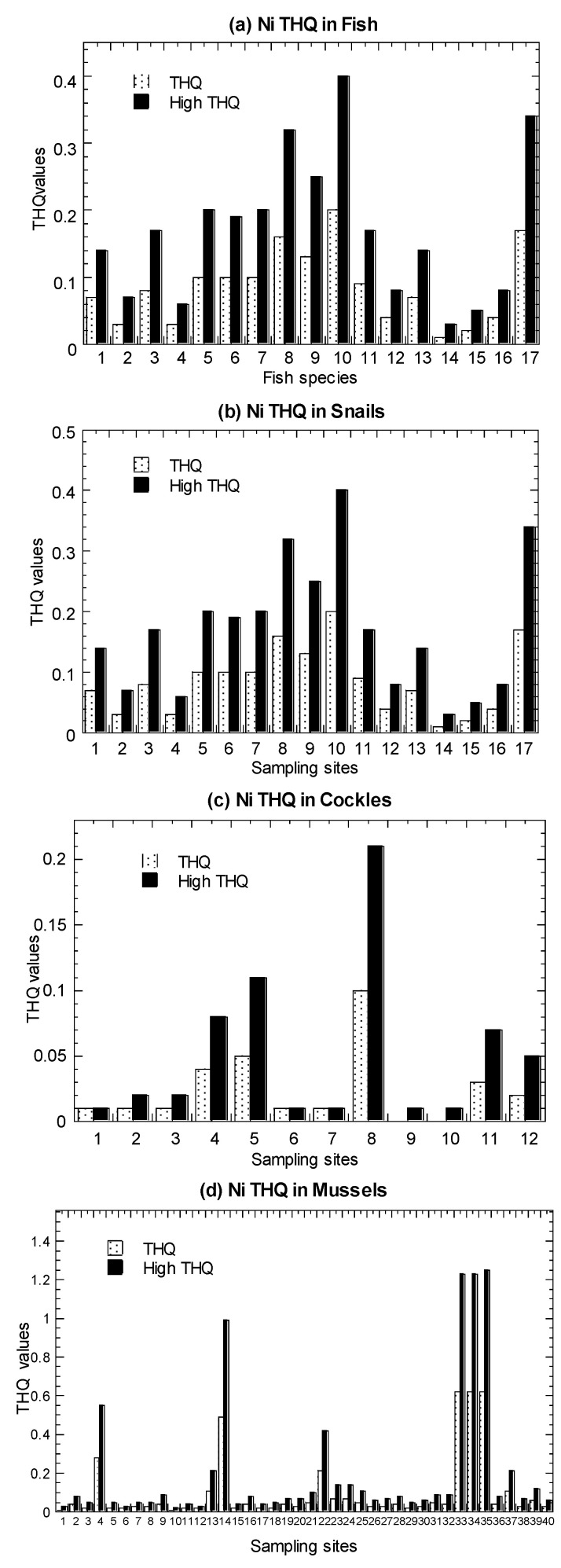
Values of average target hazard quotient (THQ) and high-level target hazard quotient (High THQ) of nickel (Ni) in the (**a**) marine fish (1–19), (**b**) mangrove snail *Cerithidea obtusa* (1–17), (**c**) cockle *Anadara granosa* (1–12), and (**d**) marine mussel *Perna viridis* (1–40) cited from Yap et al. [35]. Note: The THQ value > 1.00 is likely to induce harmful health consequences [63]. The high-level consumers (2 times of consumption rate of average-level consumers) are indicated by High.

Values of average THQ and high-level THQ in the marine fish, mangrove snails *C. obtusa*, and cockle *A. granosa*, are presented in Figure 4 and summarized in Table 1 (Tables S4–S6). 

In both the average and high-level consumers, for fish, they were found to have THQ values ranging from 0.01–0.15. Based on eight marine species (among the 19 species in Setiu samples) (Tables S9 and S10), the THQ values for average-level consumers ranged from 0.0006–2.60. For *C. obtusa*, the THQ values ranged from 0.01–0.40. For *A. granosa*, the THQ values ranged from 0.005–0.21. 

For comparative purpose, the recalculated THQ values in *P. viridis* ranged from 0.01–1.25. These THQ values were lower than 1.00, except for the high-level consumers of mussel samples collected from the polluted sites in Kampung Pasir Puteh [35]. Therefore, the THQ values for the average-level consumers of fish, snail, cockle, and mussels indicated a low non-carcinogenic risk of Ni and considered to be safe for human consumption. This also shows the absence of public health hazard of Ni risk except for the high-level consumers of mussel samples collected from the polluted sites.

Based on the different consumption rates, Jovic and Stankovic [68] also stated THQ values of <1 from Albanian mussel *Mytilus galloprovincialis*, respectively, for average-level mussel and high-level mussel consumers. In Malaysia, Cheng and Yap [87] reported that, based on mangrove snails *Nerita lineata*, all THQ values were <1 for eight trace metals (As, Cd, Cu, Cr, Fe, Hg, Pb, and Zn). 

### 3.3. Comparisons between Estimated Weekly Intake (EWI) and Provisional Tolerable Weekly Intake (PTWI)

**Figure 5:**
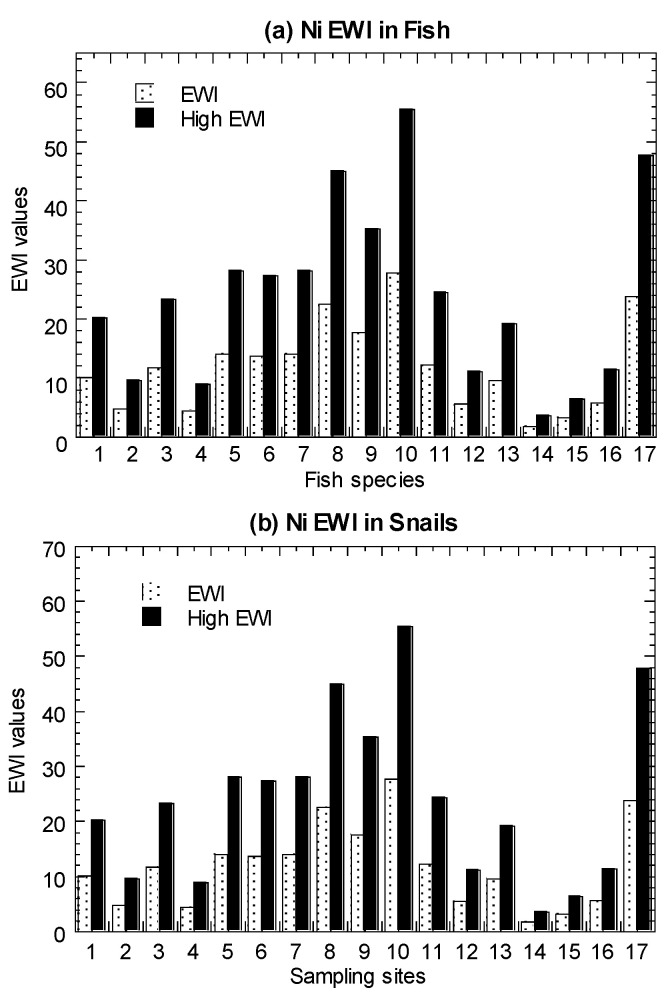

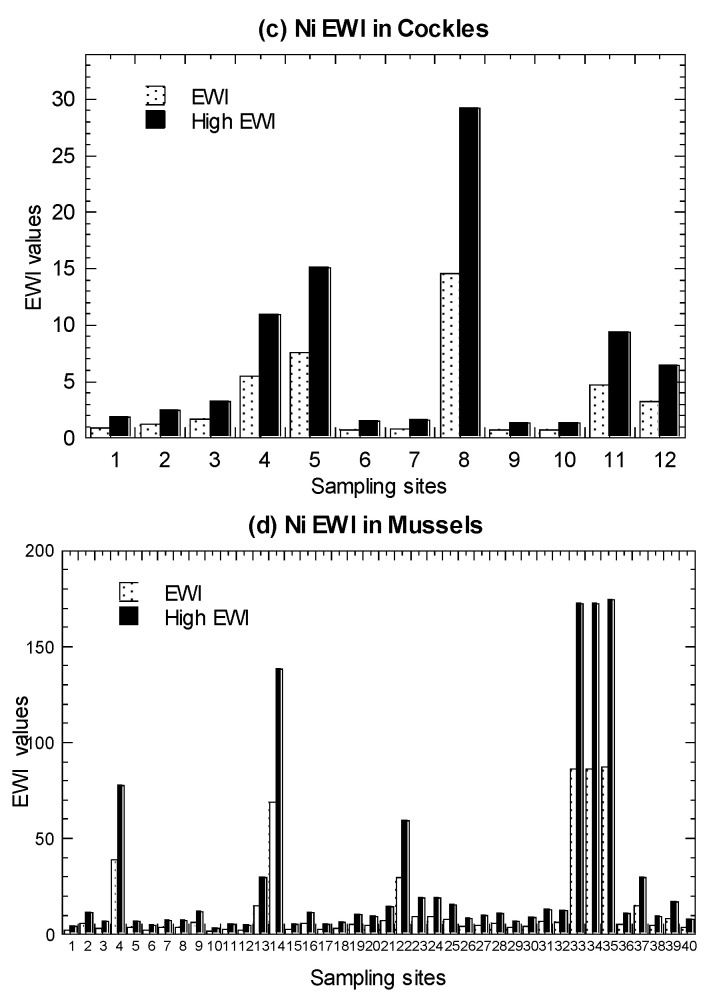
Values of average estimated weekly intake (EWI) and high-level estimated weekly intake (High EWI) of nickel (Ni) in the (**a**) marine fish (1–19), (**b**) mangrove snail *Cerithidea obtusa* (1–17), (**c**) cockle *Anadara granosa* (1–12), and (**d**) marine mussel *Perna viridis* (1–40) cited from Yap et al. [35]. Note: With provisional tolerable weekly intake (PTWI) for Ni as 91 µg/kg body weight/week [67], the Ni PTWI for 62 kg body weight for an average adult in Malaysia is equivalent to 5642 µg/week. The high-level consumers (2 times of consumption rate of average-level consumers) are indicated by High.

Values of average EWI and high-level EWI in the marine fish, *C. obtusa*, and *A. granosa*, are presented in Figure 5 and summarized in Table 1 (Tables S4–S6). In both the average and high-level consumers, fish were found to have EWI values ranging from 1.24–20.3 µg/week. For *C. obtusa*, the EWI values ranged from 1.81–55.5 µg/week. For *A. granosa*, the EWI values ranged from 0.68–29.2 µg/week. For comparative purpose, the re-calculated EWI values ranged from 1.49–174 µg/week in *P. viridis*, which are higher than those in fish, snails, and cockles of the present study (Table S7).

The results showed that the calculated EWI values in the four seafood types with average and high-level consumers were well below the calculated PTWI of Ni (5642 µg/week). Therefore, seafood consumption was not considered to have adverse effects of Ni to consumers based on the FAO/WHO JECFA guidelines. Based on nine heavy metals in 46 marine fish species from the coastal waters of Peninsular Malaysia, Azmi et al. [53] reported that the estimated Ni EWI ranged from 5.12 and 103 (µg/kg bw/week). Based on 17 reported publications of Ni in marine fishes, the Ni EWI ranged from 0.08 to 364 (µg/kg bw/week), with *Scomberomorus commerson* collected from Zhongsha (Macclesfield) Fishing Ground, South China Sea [83], being the highest. Based on eight marine species (among the 19 species in Setiu samples) (Tables S9 and S10), the EWI values for average-level consumers ranged from 0.08–364.

In the present study, the amount (kg) of samples that would need to be consumed per week by a 62 kg adult to reach the EFSA [67]’s recalculated PTWI limit (5642 µg/week) for Ni are 6.27–51.3 kg for Setiu fish, 0.92–14.1 kg for snails, 1.75–37.6 kg for cockles, 0.29–17.0 kg for mussels. In other words, if a batch of *A. maculatus* from Setiu with a Ni concentration of 0.90 mg/kg WW, a 62 kg adult could consume 6.27 kg per week of the fish without any health risk of Ni. Similarly, if a batch of *C. obtusa* from Belanak (Juru) with a Ni concentration of 5.28 mg/kg WW, a 62 kg adult could consume 1.07 kg per week of the snails without any health risk of Ni. Likewise, if a batch of *A. granosa* from Pantai Jeram with a Ni concentration of 3.23 mg/kg WW, a 62 kg adult could consume 1.75 kg per week of the snails without any health risk of Ni. Yap et al. [35] reported that the amount (kg) of mussels that would need to be consumed per week by a 60-kg adult to reach JECFA limits varied from 0.11–6.37 kg for Ni (based on JECFA (2010) established Ni PTWI of 35 µg/week/kg bw), and thus, the Ni PTWI for a 60-kg adult was equivalent to 2100 µg/week for Ni. Jovic and Stankovic [68] documented that consumers could intake 6.00 kg per week with the Ni mean of 0.35 mg/kg WW from Croatia.

In the present study, the mean weekly intake (MWI) (mg/week) of Ni for average-level (0.70 kg) consumers (mg/week) of 19 fish species from Setiu is 0.08–0.64 (high-level consumers: 0.15–1.26), and the percentages of EFSA [67]’s recalculated PTWI limit (5642 µg/week based on a 62 kg adult) of Ni for average-level consumers are 1.36–11.2% (high-level consumers: 2.73–22.3%). For *C. obtusa*, the mean weekly intake (MWI; mg/week) of Ni for average-level (0.28 kg) consumers (mg/week) of the snails is 0.11–1.72 (high-level consumers: 0.22–3.44), and the percentages of EFSA [67]’s recalculated PTWI limit (5642 µg/week based on a 62 kg adult) of Ni for average-level consumers is 1.99–30.5% (high-level consumers: 3.97–60.9%). For *A. granosa*, the mean weekly intake (MWI; mg/week) of Ni for average-level (0.28 kg) consumers (mg/week) of the cockles is 0.04–0.90 (high-level consumers: 0.08–1.81), and the percentages of EFSA [67]’s recalculated PTWI limit (5642 µg/week based on a 62 kg adult) of Ni for average-level consumers are 0.74–16.0% (high-level consumers: 1.49–32.1%) (Table 1). 

For comparative purpose, the re-calculated mean weekly intake (MWI; mg/week) of Ni in *P. viridis*, for average-level (0.28 kg) consumers (mg/week) of mussels is 0.09–5.40 (high-level consumers: 0.18–10.8), and the percentages of EFSA [67]’s recalculated PTWI limit (5642 µg/week based on a 62 kg adult) of Ni for average-level (PTWI) consumers are 1.64–95.8% (high-level consumers: 3.28–192%). The MWI of mussels are lower than that for fish but comparable to snails and cockles of the present study (Table 1). 

Based on JECFA (2010), established Ni PTWI of 35 µg/week/kg bw, and with the Ni PTWI for a 60-kg adult, Yap et al. [35] reported the Ni MWI for average-level mussel (0.125 kg) consumers and the percentage of prescribed PTWI values were 0.04–2.41 mg Ni/week and 1.96–115%, respectively. Likewise, Jovic and Stankovic [68] reported that based on the measured Ni concentrations in mussels from the Adriatic Sea, the weekly intake of Ni was estimated from 0.04–0.10 mg/person/week and from 0.09–0.21 mg/person/week for average-level mussel consumers. This accounted for 2.08–4.94% of the prescribed PTWI for Ni.

The consumption rate of seafood can greatly determine the THQ values, as seen in the present study’s high-level consumers, especially in mussels. The consumption rates of different countries for fish and shellfish differed significantly. For example, Liu et al. [88] reported the THQ values for Ni in the seasnails, bivalves, and fish were below 1.00, collected from Xiangshan Bay, China. For the Chinese consumers, molluscs, and fish consumption rates were 17.1 and 105 g/person/day, respectively [88]. They reported the Ni concentration (mg/kg WW) ranges were 0.07–1.72, 0.01–0.85, and 0.001–0.02 for seasnails, bivalves, and fish, respectively. Peycheva et al. [66] reported Ni concentrations of 0.19–0.64 mg/kg WW from wild and farmed *Mytilus galloprovincialis* from the Black Sea (Bulgaria). However, they used a relatively low average daily consumption rate of mussels (0.8 g/person/day). Therefore, comparisons of Ni THQ values between the present study and those by Liu et al. [65] and Peycheva et al. [66] are incomparable due to significant consumption rates. 

Guy et al. [21] studied the rate of shellfish consumption in New Zealand and reported an average daily consumption of 4.8 g/person/day for shellfish consumption. Nguyen et al. [89] studied the consumption rate of shellfish in Vietnam and found that mean consumption rates of the bivalves (green mussels) and gastropods were 39.3 and 16.4 g/person/day, respectively. The bivalve consumption rate was close to the present consumption rate for molluscs (40 g/person/day) from the present study. Jovic and Stankovic [68] used 17.86 and 35.7 g/person/day for average and high-level mussel consumers for the THQ estimation in the Albanian mussel *Mytilus galloprovincialis*.

Ni is a physiologically and nutritionally important trace element found in many kinds of seafood. Caution should be exercised in which this risk assessment needs more attention because Ni enters into the human body through other sources, mainly through other dietary ingestible sources such as drinking water, rice, fruits and vegetables, inhalation, and dermal contact exposure pathways [65]. There is always a potential health risk of Ni exposure that could go beyond the estimation of THQ and EWI, finally reaching the Ni PTWI value. This issue should be carefully addressed in future studies. Nonetheless, the 19 fish species from Setiu, *C. obtusa*, *A. granosa*, and *P. viridis* from Peninsular Malaysia have a low impact on consumers’ health in terms of Ni concentrations. 

## 4. Conclusions

In conclusion, the Ni concentrations in the 19 marine species of fish from Setiu, snail *C. obtusa*, and cockle *A. granosa,* were below the MPL for Ni. The HHR of Ni indicated that THQ for all three seafood is also below 1, which means that there was no non-carcinogenic risk of Ni for average and high-level consumers. It was found that the calculated values of Ni EWI were below than PTWI of Ni.

Still, following the recommended control plans is essential to reduce the health risk associated with the oral ingestion of Ni via prolonged consumption of marine fishes and molluscs. This study provided a scientific basis for the food safety assessment of Ni and suggestions for risk management of seafood consumption in Malaysia. Lastly, in the future, regular monitoring of Ni in the commercial marine fishes and molluscs should be carried out to check for food safety by assessing the health risks of PTMs to the consumers. 

## Figures and Tables

**Table 1:** Comparison of overall statistics of the values of nickel concentrations (mg/kg in dry weight (DW) and wet weight (WW)), estimated daily intake (EDI), target hazard quotient (THQ), estimated weekly intake (EWI), amount (kg) of samples that would need to be consumed per week (PTWI), weekly intake (Intake), and the percentages of EFSA [67]’s recalculated PTWI limit (PTWI%), for average and high-level consumers of nickel in the marine fish, mangrove snail *Cerithidea obtusa*, cockle *Anadara granosa*, and marine mussel *Perna viridis*. The high-level consumer (High) was based on two times the consumption rate of the average-level consumer.

Fish (N = 19)	DW	WW	EDI	High EDI	THQ	High THQ	EWI	High EWI	PTWI *	Intake *	High Intake **	PTWI% *	High PTWI% **
Minimum	0.48	0.11	0.18	0.35	0.01	0.02	1.24	2.48	6.27	0.08	0.15	1.36	2.73
Maximum	3.84	0.90	1.45	2.90	0.07	0.15	10.2	20.3	51.3	0.63	1.26	11.2	22.3
Mean	1.45	0.32	0.52	1.04	0.03	0.05	3.64	7.28	23.2	0.23	0.45	4.00	8.01
Median	1.52	0.33	0.53	1.06	0.03	0.05	3.73	7.45	17.1	0.23	0.46	4.09	8.19
SD	0.82	0.18	0.30	0.59	0.02	0.03	2.08	4.16	12.9	0.13	0.26	2.29	4.57
Snails (N = 17)	DW	WW	EDI	High EDI	THQ	High THQ	EWI	High EWI	PTWI *	Intake *	High Intake **	PTWI% *	High PTWI% **
Minimum	1.67	0.40	0.26	0.52	0.01	0.03	1.81	3.61	0.92	0.11	0.22	1.99	3.97
Maximum	25.6	6.14	3.96	7.92	0.20	0.40	27.7	55.46	14.11	1.72	3.44	30.5	60.9
Mean	10.9	2.64	1.70	3.40	0.08	0.17	11.9	23.82	3.63	0.74	1.48	13.1	26.2
Median	10.7	2.58	1.66	3.33	0.08	0.17	11.6	23.30	2.19	0.72	1.44	12.8	25.6
SD	7.00	1.68	1.08	2.17	0.06	0.11	7.59	15.18	3.34	0.47	0.94	8.34	16.7
Cockles (N = 12)	DW	WW	EDI	High EDI	THQ	High THQ	EWI	High EWI	PTWI *	Intake *	High Intake **	PTWI%*	High PTWI%**
Minimum	0.74	0.15	0.10	0.19	0.00	0.01	0.68	1.35	1.75	0.04	0.08	0.74	1.49
Maximum	16.2	3.23	2.08	4.17	0.10	0.21	14.6	29.2	37.6	0.90	1.81	16.0	32.1
Mean	3.88	0.78	0.50	1.00	0.02	0.05	3.51	7.01	19.3	0.22	0.44	3.85	7.71
Median	1.58	0.32	0.20	0.41	0.01	0.02	1.43	2.85	18.3	0.09	0.18	1.57	3.13
SD	4.62	0.92	0.59	1.19	0.03	0.06	4.17	8.34	14.5	0.26	0.52	4.58	9.16
Mussels (N = 40)	DW	WW	EDI	High EDI	THQ	High THQ	EWI	High EWI	PTWI *	Intake *	High Intake **	PTWI% *	High PTWI% **
Minimum	1.94	0.33	0.21	0.43	0.01	0.02	1.49	2.98	0.29	0.09	0.18	1.64	3.28
Maximum	114	19.3	12.5	24.9	0.62	1.25	87.2	174	17.1	5.40	10.81	95.8	191
Mean	18.9	3.2	2.07	4.13	0.10	0.21	14.5	28.9	5.42	0.90	1.79	15.9	31.8
Median	6.91	1.18	0.76	1.52	0.04	0.07	5.30	10.6	4.80	0.33	0.66	5.84	11.7
SD	31.5	5.33	3.44	6.87	0.17	0.34	24.1	48.1	3.72	1.49	2.98	26.4	52.9

Note SD = standard deviation; PTWI * = amount (kg) of samples that would need to be consumed per week by a 62 kg adult to reach the EFSA [67]’s recalculated PTWI limit (5642 µg/week) for nickel. Intake * = weekly intake (MWI; mg/week) of nickel for average-level fish (0.70 kg) and mollusc (0.28 kg) consumers (mg/week) and the percentages of EFSA [67]’s recalculated PTWI limit (5642 µg/week based on a 62 kg adult) of nickel for average-level (PTWI%*) consumers. Intake ** = mean weekly intake (MWI; mg/week) of nickel for high-level fish (1.40 kg) and mollusc (0.56 kg) consumers (mg/week) and the percentages of EFSA [67]’s recalculated PTWI limit (5642 µg/week based on a 62 kg adult) of nickel for high-level (PTWI%**) consumers.

## Data Availability

Not applicable.

## Supplementary Materials

The following supporting information can be downloaded at: https://www.mdpi.com/article/10.3390/biology11030376/s1, Table S1. Marine fishes collected from fishing loading sites at Kampong Fikri (1) and Kampung Rhu Sepuluh (2) in Setiu, Terengganu, Peninsular Malaysia; Table S2. Sampling details of cockle *Anadara granosa* from the west coast of Peninsular Malaysia; Table S3. Sampling details of mangrove snail *Cerithidea obtusa* from the west coast of Peninsular Malaysia; Table S4. The values of nickel concentrations (mg/kg in dry weight) and wet weight, average estimated daily intake, target hazard quotient, estimated weekly intake, amount (kg) of samples that would need to be consumed per week by a 62 kg adult to reach the EFSA [67]’s (PTWI), mean weekly intake (mg/week) of nickel and the percentages of EFSA [67]’s recalculated PTWI limit (5642 µg/week based on a 62 kg adult) of nickel (PTWI%*), for average-level consumers, in the 19 species of marine fishes collected from Setiu, Terengganu, Peninsular Malaysia; Table S5. The values of nickel concentrations (mg/kg in dry weight and wet weight), average estimated daily intake, target hazard quotient, estimated weekly intake, amount (kg) of samples that would need to be consumed per week by a 62 kg adult to reach the EFSA [67]’s PTWI, mean weekly intake (mg/week) of nickel and the percentages of EFSA [67]’s recalculated PTWI limit (5642 µg/week based on a 62 kg adult) of nickel (PTWI%*), for average-level consumers, in the 17 populations of mangrove snail *Cerithidea obtusa* collected from the west coast mangrove of Peninsular Malaysia; Table S6. The values of nickel concentrations (mg/kg in dry weight and wet weight), average estimated daily intake, target hazard quotient, estimated weekly intake, amount (kg) of samples that would need to be consumed per week by a 62 kg adult to reach the EFSA [2]’s PTWI, mean weekly intake (mg/week) of nickel and the percentages of EFSA [67]’s recalculated PTWI limit (5642 µg/week based on a 62 kg adult) of nickel (PTWI%*), for average-level consumers, in the 12 populations of cockle *Anadara granosa* collected from the west intertidal area of Peninsular Malaysia; Table S7. The values of nickel concentrations (mg/kg in dry weight and wet weight), recalculated values of average estimated daily intake, target hazard quotient, estimated weekly intake, amount (kg) of samples that would need to be consumed per week by a 62 kg adult to reach the EFSA [67]’s PTWI, mean weekly intake (mg/week) of nickel and the percentages of EFSA [67]’s recalculated PTWI limit (5642 µg/week based on a 62 kg adult) of nickel (PTWI% *), for average-level consumers, in the 40 populations of mussel *Perna viridis* from the coastal waters of Peninsular Malaysia (Yap et al. [35]); Table S8. Mean nickel concentrations (mg/kg dry weight and wet weight) in various species of marine fishes reported in the literature, including the results of the present study (8 species); Table S9. Nickel values of estimated daily intake, target hazard quotient, and estimated weekly intake (EWI) calculated based on the present study and cited Ni data in the marine fishes from the literature; Table S10: Overall statistics of nickel concentrations (mg/kg wet weight) with recalculation of estimated daily intake, target hazard quotient, and estimated weekly intake in the 8 marine fish species cited from the literature.
