# Supplementary material for: Comparative Study of Potentially Toxic Nickel and Their Potential Human Health Risks in Seafood (Fish and Mollusks) from Peninsular Malaysia
Source: Biology (Basel). 2022 Feb 27;11(3):376. doi: 10.3390/biology11030376 (PMC8945417; doi:10.3390/biology11030376)
Supplement: Supplementary file 1 [file biology-11-00376-s001.zip › biology-1564712-supplementary.pdf]

<u>Supplementary Materials</u>

Comparative study of potentially toxic nickel and their potential human health risks in the seafood (fish and mollusks) from Peninsular Malaysia

Chee Kong Yap[1*], and Khalid Awadh Al-Mutairi[2]

[1]Department of Biology, Faculty of Science, Universiti Putra Malaysia, 43400 UPM Serdang, Malaysia;
[2]Department of Biology, Faculty of Science, University of Tabuk, Tabuk 741, Saudi Arabia;
*Corresponding author: yapckong@hotmail.com; yapchee@upm.edu.my

Table S1. Marine fishes collected from fishing loading sites at Kampong Fikri (1) and Kampung Rhu Sepuluh (2) in Setiu, Terengganu, Peninsular Malaysia.
Table S2. Sampling details of cockle *Anadara granosa* from the west coast of Peninsular Malaysia.
Table S3. Sampling details of mangrove snail *Cerithidea obtusa* from the west coast of Peninsular Malaysia.
Table S4. The values of nickel concentrations (mg/kg in dry weight (DW) and wet weight (WW)), average estimated daily intake (EDI), target hazard quotient (THQ), estimated week intake (EWI), amount (kg) of samples that would need to be consumed per week by a 62-kg adult to reach the EFSA [2])'s (PTWI), mean weekly intake (MWI; mg/week) of nickel and the percentages of EFSA [2]'s recalculated PTWI limit (5642 µg/week based on a 62-kg adult) of nickel (PTWI%*), for average-level consumers, in the 19 species of marine fishes collected from Setiu, Terengganu, Peninsular Malaysia. The high-level consumers (2 times of consumption rate of average-level consumers) are indicated by High.
Table S5. The values of nickel concentrations (mg/kg in dry weight (DW) and wet weight (WW)), average estimated daily intake (EDI), target hazard quotient (THQ), estimated week intake (EWI), amount (kg) of samples that would need to be consumed per week by a 62-kg adult to reach the EFSA [2])'s (PTWI), mean weekly intake (MWI; mg/week) of nickel and the percentages of EFSA [2]'s recalculated PTWI limit (5642 µg/week based on a 62-kg adult) of nickel (PTWI%*), for average-level consumers, in the 17 populations of mangrove snail *Cerithidea obtusa* collected from the west coast mangrove of Peninsular Malaysia. The high-level consumers (2 times of consumption rate of average-level consumers) are indicated by High.
Table S6. The values of nickel concentrations (mg/kg in dry weight (DW) and wet weight (WW)), average estimated daily intake (EDI), target hazard quotient (THQ), estimated week intake (EWI), amount (kg) of samples that would need to be consumed per week by a 62-kg adult to reach the EFSA [2])'s (PTWI), mean weekly intake (MWI; mg/week) of nickel and the percentages of EFSA [2]'s recalculated PTWI limit (5642 µg/week based on a 62-kg adult) of nickel (PTWI%*), for average-level consumers, in the 12 populations of cockle *Anadara granosa* collected from the west intertidal area of Peninsular Malaysia. The high-level consumers (2 times of consumption rate of average-level consumers) are indicated by High.
Table S7. The values of nickel concentrations (mg/kg in dry weight (DW) and wet weight (WW)), recalculated values of average estimated daily intake (EDI), target hazard quotient (THQ), estimated week intake (EWI), amount (kg) of samples that would need to be consumed per week by a 62-kg adult to reach the EFSA [2])'s (PTWI), mean weekly intake (MWI; mg/week) of nickel and the percentages of EFSA [2]'s recalculated PTWI limit (5642 µg/week based on a 62-kg adult) of nickel (PTWI%*), for average-level consumers, in the 40 populations of mussel *Perna viridis* from the coastal waters of Peninsular Malaysia (Yap et al. [1]). The high-level consumers (2 times of consumption rate of average-level consumers) are indicated by High.
Table S8. Mean nickel concentrations (mg/kg dry weight (DW) and wet weight (WW)) in various species of marine fishes reported in the literature, including the results of the present study (8 species).
Table S9. Nickel; values of estimated daily intake (EDI), target hazard quotient (THQ), and estimated weekly intake (EWI) calculated based on the present study and cited Ni data in the marine fishes from the literature.
Table S10: Overall statistics of nickel concentrations (mg/kg wet weight) (WW) with recalculation of estimated daily intake (EDI), target hazard quotient (THQ), and estimated weekly intake (EWI) in the 8 marine fish species cited from the literature (17 reports of 17 papers) (N= 17).

Table S1. Marine fishes collected from fishing loading sites at Kampong Fikri (1) and Kampung Rhu Sepuluh (2) in Setiu, Terengganu, Peninsular Malaysia.

Site	Common name	Scientific name	N	Conversion factor	Habitat niche
		Family: *Carangidae*			Pelagic fish
1	Malabar trevally	*Carangoides malabaricus*	5	0.24	Reef-associated
1	Yellowtail scad	*Atule mate*	5	0.23	Reef-associated
1	Shortfin scad	*Decapterus macrosoma*	5	0.20	Reef-associated
1	Yellow stripe scad	*Selaroides leptolepis*	5	0.24	Reef-associated
1	Torpedo scad	*Megalaspis cordyla*	5	0.23	Reef-associated
1	Indian threadfish	*Alectis indica*	3	0.21	Reef-associated
		Family: Stromateidae			
1	Chinese silver pomfret	*Pampus chinensis*	3	0.19	Benthopelagic
		Family: Clupeidae			
1	Chacunda gizzard shard	*Anodontostoma chacunda*	5	0.23	Pelagic-neritic
		Family: Chirocentridae			
1	Dorab wolf-herring	*Chirocentrus dorab*	5	0.23	Reef-associated
		Family: *Scrombidae*			Pelagic
1	Indian mackerel	*Rastrelliger kanagurta*	5	0.26	Pelagic-neritic
1	Narrow-barred Spanish mackerel	*Scomberomorus commerson*	3	0.23	Pelagic-neritic
		Family: *Dasyatidae*			
1	Dwarf whipray	*Himantura walga*	3	0.21	Demersal
		Family: Nemipteridae			
1	Ornate threadfin bream	*Nemipterus hexodon*	5	0.20	Demersal
		Family: Lactariidae			
1	False trevally	*Lactarius lactarius*	5	0.21	Pelagic-neritic
		Family: Trichiuridae			
1	Large head hairtail	*Trichiurus lepturus*	3	0.22	Benthopelagic
		Family: Ariidae			
1	Spotted catfish	*Arius maculatus*	3	0.23	Demersal
		Family: Sciaenidae			
2	Tigertooth croaker	*Otolithes ruber*	3	0.24	Benthopelagic
2	Goatee croaker	*Dendrophysa russelli*	5	0.23	Demersal
2	Belanger's croaker	*Johnius belangerii*	3	0.23	Demersal

Note: Conversion factor is wet weight (g) which was divided by dry weight (g) of the sample.

Table S2. Sampling details of cockle *Anadara granosa* from the west coast of Peninsular Malaysia.

Site no.	Sampling sites	Sampling dates	Lat (N)	Long (E)
1	Bayan Lepas, Penang (BLepas)	5 Oct 2008	5°16'60.00"	100°16'0.00"
2	Batu Melintang. Perak (BMelintang)	5 Nov 2008	5°37′ 21.58″	100°24′ 06.18″
3	Kuala Juru-1, Penang (Juru-1)	5 Oct 2008	5°19'41.40"	100°23'1.44"
4	Kuala Juru-2, Penang (Juru-2)	20 Apr 2005	100°26.083'	05°19.772'
5	Kuala Juru-3, Penang (Juru-3)	8 Dec 2007	5°20'6.59"	100°24'3.98"
6	Kuala Kurau Jetty, Perak (KKurau)	19 Apr 2005	100°25.867'	05°00.928'
7	Minyak Beku, Johor (Mbeku)	5 Feb 2008	01°, 45.315′	102°, 55.480′
8	Pantai Jeram, Selangor (Jeram)	25 Apr 2005	03°10.403′	101°18.819′
9	Sg. Bharu, Penang (SBahru)	5 Nov 2008	6°19'53.40"	100°09'24.82"
10	Sg. Ayam, Johor (SAyam)	5 Feb 2008	01° 45.289′	102° 55.873′
11	Sg. Belanak, Penang (SBelanak)	7 Dec 2007	5°18'14.56"	100°24'50.37"
12	Sg. Klang-1, Selangor (Klang)	2 Dec 2007	101°22.511′	03°01.343′,

Table S3. Sampling details of mangrove snail *Cerithidea obtusa* from the west coast of Peninsular Malaysia.

Site no.	Sampling sites	Sampling dates	Lat (N)	Long (E)
1	Sepang Besar-1, Selangor	1 Dec 2007	02°36.653'	101°42.345'
2	Sepang Besar-2, Selangor	1 Dec 2007	02°38.933	101°42.826'
3	Klang-1, Selangor	2 Dec 2007	03°01.343′	101°22.511′
4	Klang-2, Selangor	2 Dec 2007	03°01.044′	101°20.560′
5	Klang-3, Selangor	2 Dec 2007	03°01.615′	101°21.150′
6	Tanjung Kupang, Johor (TKupang)	19 Jun 2010	1°22.694"	103°38.094"
7	Sg. Ayam, Johor (SAyam)	18 Jun 2010	2°34.423"	102°02.346"
8	Sepang Besar, Selangor	5 May 2010	2°36.230"	101°42.139"
9	Lukut-1, Negeri Sembilan	27 Dec 2010	2°35.153"	101°49.548"
10	Lukut-2, Negeri Sembilan	27 Dec 2010	2°35.958"	101°47.509"
11	Kg. Deralik, Perak (Deralik)	25 Feb 2006	04°14′53.8″	100°42′ 09.1″
12	Laut Kurong Tengar (LKTengar)	5 Dec 2008	6°22'19.29"N	10 ° 8'32.10"E
13	Muar River, Johor (Muar)	8 May 2007	02°09.573′	102°19.033′
14	Lukut-1, Negeri Sembilan	1 Dec 2007	02°34.978' ,	101°47.607'
15	Lukut-2, Negeri Sembilan	1 Dec 2007	02°34.847' ,	101°47.795'
16	Lukut-3, Negeri Sembilan	1 Dec 2007	02°34.853' ,	101°47.882'
17	Sg. Belanak-1, Penang (SBelanak)	7 Dec 2007	5°18'14.56"	100°24'50.37"

Table S4. The values of nickel concentrations (mg/kg in dry weight (DW) and wet weight (WW)), average estimated daily intake (EDI), target hazard quotient (THQ), estimated week intake (EWI), amount (kg) of samples that would need to be consumed per week by a 62-kg adult to reach the EFSA [2])'s (PTWI), mean weekly intake (MWI; mg/week) of nickel and the percentages of EFSA [2]'s recalculated PTWI limit (5642 µg/week based on a 62-kg adult) of nickel (PTWI%*), for average-level consumers, in the 19 species of marine fishes collected from Setiu, Terengganu, Peninsular Malaysia. The high level consumers (2 times of consumption rate of average-level consumers) are indicated by High.

No	Marine fish species	DW	WW	EDI	THQ	EWI	High EDI	High THQ	High EWI	Amount PTWI	Intake*	High Intake**	PTWI%*	High PTWI%**
1	*Carangoides malabaricus*	1.52	0.37	0.60	0.03	4.18	1.19	0.06	8.35	15.25	0.26	0.52	4.59	9.18
2	*Atule mate*	0.64	0.15	0.24	0.01	1.69	0.48	0.02	3.39	37.61	0.11	0.21	1.86	3.72
3	*Decapterus macrosoma*	0.65	0.13	0.21	0.01	1.47	0.42	0.02	2.94	43.40	0.09	0.18	1.61	3.23
4	*Selaroides leptolepis*	0.64	0.16	0.26	0.01	1.81	0.52	0.03	3.61	35.26	0.11	0.22	1.99	3.97
5	*Megalaspis cordyla*	0.48	0.11	0.18	0.01	1.24	0.35	0.02	2.48	51.29	0.08	0.15	1.36	2.73
6	*Otolithes ruber*	1.16	0.28	0.45	0.02	3.16	0.90	0.05	6.32	20.15	0.20	0.39	3.47	6.95
7	*Dendrophysa russelli*	1.56	0.35	0.56	0.03	3.95	1.13	0.06	7.90	16.12	0.25	0.49	4.34	8.68
8	*Johnius belangeri*	1.14	0.26	0.42	0.02	2.94	0.84	0.04	5.87	21.70	0.18	0.36	3.23	6.45
9	*Pampus chinensis*	1.71	0.33	0.53	0.03	3.73	1.06	0.05	7.45	17.10	0.23	0.46	4.09	8.19
10	*Anodontostama chacunda*	1.93	0.44	0.71	0.04	4.97	1.42	0.07	9.94	12.82	0.31	0.62	5.46	10.92
11	*Chirocentrus dorab*	1.44	0.33	0.53	0.03	3.73	1.06	0.05	7.45	17.10	0.23	0.46	4.09	8.19
12	*Rastrelliger kanagurta*	0.64	0.16	0.26	0.01	1.81	0.52	0.03	3.61	35.26	0.11	0.22	1.99	3.97
13	*Himantura walga*	0.65	0.14	0.23	0.01	1.58	0.45	0.02	3.16	40.30	0.10	0.20	1.74	3.47
14	*Nemipterus hexodon*	1.93	0.38	0.61	0.03	4.29	1.23	0.06	8.58	14.85	0.27	0.53	4.71	9.43
15	*Alectis indica*	2.27	0.47	0.76	0.04	5.31	1.52	0.08	10.61	12.00	0.33	0.66	5.83	11.66
16	*Lactarius lactarius*	1.52	0.32	0.52	0.03	3.61	1.03	0.05	7.23	17.63	0.22	0.45	3.97	7.94
17	*Scomberomorus commerson*	1.52	0.35	0.56	0.03	3.95	1.13	0.06	7.90	16.12	0.25	0.49	4.34	8.68
18	*Trichiurus lepturus*	2.29	0.50	0.81	0.04	5.65	1.61	0.08	11.29	11.28	0.35	0.70	6.20	12.41
19	*Arius maculatus*	3.84	0.90	1.45	0.07	10.16	2.90	0.15	20.32	6.27	0.63	1.26	11.17	22.33

Note: The dry weight basis was converted into wet weight by using the respective conversion factor of each fish species, as shown in Table 1.
Amount PTWI= Amount (kg) of samples that would need to be consumed per week by a 62-kg adult to reach the EFSA [2]'s recalculated PTWI limit (5642 µg/week) for nickel.
Intake*= Mean weekly intake (MWI; mg/week) of nickel for average level fish (0.70 kg) and molluscs (0.28 kg) consumers (mg/week) and the percentages of EFSA [2]'s recalculated PTWI limit (5642 µg/week based on a 62-kg adult) of nickel for average level (PTWI%*) consumers.
Intake**= Mean weekly intake (MWI; mg/week) of nickel for high-level fish (1.40 kg) and molluscs (0.56 kg) consumers (mg/week) and the percentages of EFSA [2]'s recalculated PTWI limit (5642 µg/week based on a 62-kg adult) of nickel for high level (PTWI%**) consumers.

Table S5. The values of nickel concentrations (mg/kg in dry weight (DW) and wet weight (WW)), average estimated daily intake (EDI), target hazard quotient (THQ), estimated week intake (EWI), amount (kg) of samples that would need to be consumed per week by a 62-kg adult to reach the EFSA [2])'s (PTWI), mean weekly intake (MWI; mg/week) of nickel and the percentages of EFSA [2]'s recalculated PTWI limit (5642 μg/week based on a 62-kg adult) of nickel (PTWI%*), for average-level consumers, in the 17 populations of mangrove snail *Cerithidea obtusa* collected from the west coast mangrove of Peninsular Malaysia. The high level consumers (2 times of consumption rate of average-level consumers) are indicated by High.

No.	Sampling sites	DW	WW	EDI	THQ	EWI	High EDI	High THQ	High EWI	Amount PTWI	Intake*	High Intake**	PTWI%*	High PTWI%**
1	SepangB-1	9.35	2.24	1.45	0.07	10.12	2.89	0.14	20.23	2.52	0.63	1.25	11.12	22.23
2	SepangB-2	4.43	1.06	0.68	0.03	4.79	1.37	0.07	9.57	5.32	0.30	0.59	5.26	10.52
3	Klang-1	10.74	2.58	1.66	0.08	11.65	3.33	0.17	23.30	2.19	0.72	1.44	12.80	25.61
4	Klang-L	4.12	0.99	0.64	0.03	4.47	1.28	0.06	8.94	5.70	0.28	0.55	4.91	9.83
5	Klang-3	12.95	3.11	2.01	0.10	14.05	4.01	0.20	28.09	1.81	0.87	1.74	15.43	30.87
6	TKupang	12.58	3.02	1.95	0.10	13.64	3.90	0.19	27.28	1.87	0.85	1.69	14.99	29.98
7	SAyam	12.94	3.11	2.01	0.10	14.05	4.01	0.20	28.09	1.81	0.87	1.74	15.43	30.87
8	SepangB	20.77	4.98	3.21	0.16	22.49	6.43	0.32	44.98	1.13	1.39	2.79	24.71	49.43
9	Lukut-1	16.31	3.91	2.52	0.13	17.66	5.05	0.25	35.32	1.44	1.09	2.19	19.40	38.81
10	Lukut-2	25.59	6.14	3.96	0.20	27.73	7.92	0.40	55.46	0.92	1.72	3.44	30.47	60.94
11	Deralik	11.25	2.7	1.74	0.09	12.19	3.48	0.17	24.39	2.09	0.76	1.51	13.40	26.80
12	LKTengar	5.11	1.23	0.79	0.04	5.55	1.59	0.08	11.11	4.59	0.34	0.69	6.10	12.21
13	Muar	8.85	2.12	1.37	0.07	9.57	2.74	0.14	19.15	2.66	0.59	1.19	10.52	21.04
14	Lukut-1	1.67	0.4	0.26	0.01	1.81	0.52	0.03	3.61	14.11	0.11	0.22	1.99	3.97
15	Lukut-2	2.95	0.71	0.46	0.02	3.21	0.92	0.05	6.41	7.95	0.20	0.40	3.52	7.05
16	Lukut-3	5.26	1.26	0.81	0.04	5.69	1.63	0.08	11.38	4.48	0.35	0.71	6.25	12.51
17	Belanak (Juru-1)	21.98	5.28	3.41	0.17	23.85	6.81	0.34	47.69	1.07	1.48	2.96	26.20	52.41

Note: The dry weight basis was converted into wet weight by using a conversion factor of 0.24.
Amount PTWI= Amount (kg) of samples that would need to be consumed per week by a 62-kg adult to reach the EFSA [2])'s recalculated PTWI limit (5642 μg/week) for nickel.
Intake*= Mean weekly intake (MWI; mg/week) of nickel for average level fish (0.70 kg) and molluscs (0.28 kg) consumers (mg/week) and the percentages of EFSA [2]'s recalculated PTWI limit (5642 μg/week based on a 62-kg adult) of nickel for average level (PTWI%*) consumers.
Intake**= Mean weekly intake (MWI; mg/week) of nickel for high-level fish (1.40 kg) and molluscs (0.56 kg) consumers (mg/week) and the percentages of EFSA [2]'s recalculated PTWI limit (5642 μg/week based on a 62-kg adult) of nickel for high level (PTWI%**) consumers.

Table S6. The values of nickel concentrations (mg/kg in dry weight (DW) and wet weight (WW)), average estimated daily intake (EDI), target hazard quotient (THQ), estimated week intake (EWI), amount (kg) of samples that would need to be consumed per week by a 62-kg adult to reach the EFSA [2])'s (PTWI), mean weekly intake (MWI; mg/week) of nickel and the percentages of EFSA [2]'s recalculated PTWI limit (5642 µg/week based on a 62-kg adult) of nickel (PTWI%*), for average-level consumers, in the 12 populations of cockle *Anadara granosa* collected from the west intertidal area of Peninsular Malaysia. The high level consumers (2 times of consumption rate of average-level consumers) are indicated by High.

No.	Sampling sites	DW	WW	EDI	THQ	EWI	High EDI	High THQ	High EWI	Amount PTWI	Intake*	High Intake**	PTWI%*	High PTWI%**
1	BLepas	1	0.2	0.13	0.01	0.90	0.26	0.01	1.81	28.21	0.06	0.11	0.99	1.99
2	BMelintang	1.35	0.27	0.17	0.01	1.22	0.35	0.02	2.44	20.90	0.08	0.15	1.34	2.68
3	KJuru-1	1.8	0.36	0.23	0.01	1.63	0.46	0.02	3.25	15.67	0.10	0.20	1.79	3.57
4	KJuru-2	6.06	1.21	0.78	0.04	5.46	1.56	0.08	10.93	4.66	0.34	0.68	6.00	12.01
5	KJuru-3	8.36	1.67	1.08	0.05	7.54	2.15	0.11	15.08	3.38	0.47	0.94	8.29	16.58
6	KKurau	0.8	0.16	0.10	0.01	0.72	0.21	0.01	1.45	35.26	0.04	0.09	0.79	1.59
7	Mbeku	0.83	0.17	0.11	0.01	0.77	0.22	0.01	1.54	33.19	0.05	0.10	0.84	1.69
8	PJeram	16.15	3.23	2.08	0.10	14.59	4.17	0.21	29.17	1.75	0.90	1.81	16.03	32.06
9	S Bahru	0.74	0.15	0.10	0.00	0.68	0.19	0.01	1.35	37.61	0.04	0.08	0.74	1.49
10	SAyam	0.76	0.15	0.10	0.00	0.68	0.19	0.01	1.35	37.61	0.04	0.08	0.74	1.49
11	SBelanak-1	5.2	1.04	0.67	0.03	4.70	1.34	0.07	9.39	5.43	0.29	0.58	5.16	10.32
12	SKlang-1	3.57	0.71	0.46	0.02	3.21	0.92	0.05	6.41	7.95	0.20	0.40	3.52	7.05

Note: The dry weight basis was converted into wet weight by using a conversion factor of 0.20.
Amount PTWI= Amount (kg) of samples that would need to be consumed per week by a 62-kg adult to reach the EFSA [2]'s recalculated PTWI limit (5642 µg/week) for nickel.
Intake*= Mean weekly intake (MWI; mg/week) of nickel for average level fish (0.70 kg) and molluscs (0.28 kg) consumers (mg/week) and the percentages of EFSA [2]'s recalculated PTWI limit (5642 µg/week based on a 62-kg adult) of nickel for average level (PTWI%*) consumers.
Intake**= Mean weekly intake (MWI; mg/week) of nickel for high-level fish (1.40 kg) and molluscs (0.56 kg) consumers (mg/week) and the percentages of EFSA [2]'s recalculated PTWI limit (5642 µg/week based on a 62-kg adult) of nickel for high level (PTWI%**) consumers.

Table S7. The values of nickel concentrations (mg/kg in dry weight (DW) and wet weight (WW)), recalculated values of average estimated daily intake (EDI), target hazard quotient (THQ), estimated week intake (EWI), amount (kg) of samples that would need to be consumed per week by a 62-kg adult to reach the EFSA [2])'s (PTWI), mean weekly intake (MWI; mg/week) of nickel and the percentages of EFSA [2]'s recalculated PTWI limit (5642 µg/week based on a 62-kg adult) of nickel (PTWI%*), for average-level consumers, in the 40 populations of mussel *Perna viridis* from the coastal waters of Peninsular Malaysia (Yap et al. [1]). The high-level consumers (2 times of consumption rate of average-level consumers) are indicated by High.

No.	Sampling sites	DW	WW	EDI	THQ	EWI	High EDI	High THQ	High EWI	Amount PTWI	Intake*	High Intake**	PTWI%*	High PTWI%**
1	Png-1	2.7	0.46	0.30	0.01	2.08	0.59	0.03	4.15	12.27	0.13	0.26	2.28	4.57
2	Png-2	7.37	1.25	0.81	0.04	5.65	1.61	0.08	11.29	4.51	0.35	0.70	6.20	12.41
3	Png-3	4.25	0.72	0.46	0.02	3.25	0.93	0.05	6.50	7.84	0.20	0.40	3.57	7.15
4	BT-1	50.5	8.58	5.54	0.28	38.75	11.07	0.55	77.50	0.66	2.40	4.80	42.58	85.16
5	PPjg-1	4.54	0.77	0.50	0.02	3.48	0.99	0.05	6.95	7.33	0.22	0.43	3.82	7.64
6	TE-1	3.08	0.52	0.34	0.02	2.35	0.67	0.03	4.70	10.85	0.15	0.29	2.58	5.16
7	TE-2	4.76	0.81	0.52	0.03	3.66	1.05	0.05	7.32	6.97	0.23	0.45	4.02	8.04
8	Merli-1	4.91	0.83	0.54	0.03	3.75	1.07	0.05	7.50	6.80	0.23	0.46	4.12	8.24
9	Sb-1	7.84	1.33	0.86	0.04	6.01	1.72	0.09	12.01	4.24	0.37	0.74	6.60	13.20
10	MBe-1	1.94	0.33	0.21	0.01	1.49	0.43	0.02	2.98	17.10	0.09	0.18	1.64	3.28
11	MBe-2	3.44	0.58	0.37	0.02	2.62	0.75	0.04	5.24	9.73	0.16	0.32	2.88	5.76
12	PJ-1	3.08	0.52	0.34	0.02	2.35	0.67	0.03	4.70	10.85	0.15	0.29	2.58	5.16
13	Seng-1	19.4	3.29	2.12	0.11	14.86	4.25	0.21	29.72	1.71	0.92	1.84	16.33	32.66
14	Kkp-1	90.1	15.3	9.87	0.49	69.10	19.74	0.99	138.19	0.37	4.28	8.57	75.93	151.86
15	Kkp-2	3.43	0.58	0.37	0.02	2.62	0.75	0.04	5.24	9.73	0.16	0.32	2.88	5.76
16	Kkp-3	7.43	1.26	0.81	0.04	5.69	1.63	0.08	11.38	4.48	0.35	0.71	6.25	12.51
17	TKup-1	3.21	0.55	0.35	0.02	2.48	0.71	0.04	4.97	10.26	0.15	0.31	2.73	5.46
18	TKup-2	4.16	0.71	0.46	0.02	3.21	0.92	0.05	6.41	7.95	0.20	0.40	3.52	7.05
19	GPt-1	6.8	1.16	0.75	0.04	5.24	1.50	0.07	10.48	4.86	0.32	0.65	5.76	11.51
20	KSMel-1	5.96	1.01	0.65	0.03	4.56	1.30	0.07	9.12	5.59	0.28	0.57	5.01	10.02
21	KSMel-2	9.29	1.58	1.02	0.05	7.14	2.04	0.10	14.27	3.57	0.44	0.88	7.84	15.68
22	KSMel-3	38.6	6.56	4.23	0.21	29.63	8.46	0.42	59.25	0.86	1.84	3.67	32.56	65.11
23	PLd-1	12.4	2.11	1.36	0.07	9.53	2.72	0.14	19.06	2.67	0.59	1.18	10.47	20.94

24	PLd-2	12.4	2.11	1.36	0.07	9.53	2.72	0.14	19.06	2.67	0.59	1.18	10.47	20.94
25	PLd-3	10	1.7	1.10	0.05	7.68	2.19	0.11	15.35	3.32	0.48	0.95	8.44	16.87
26	PLd-4	5.53	0.94	0.61	0.03	4.25	1.21	0.06	8.49	6.00	0.26	0.53	4.67	9.33
27	Senib-1	6.3	1.07	0.69	0.03	4.83	1.38	0.07	9.66	5.27	0.30	0.60	5.31	10.62
28	Senib-2	7.12	1.21	0.78	0.04	5.46	1.56	0.08	10.93	4.66	0.34	0.68	6.00	12.01
29	Senib-3	4.45	0.76	0.49	0.02	3.43	0.98	0.05	6.86	7.42	0.21	0.43	3.77	7.54
30	TJ-1	5.64	0.96	0.62	0.03	4.34	1.24	0.06	8.67	5.88	0.27	0.54	4.76	9.53
31	Masai-1	8.46	1.44	0.93	0.05	6.50	1.86	0.09	13.01	3.92	0.40	0.81	7.15	14.29
32	Masai-1	8.14	1.38	0.89	0.04	6.23	1.78	0.09	12.46	4.09	0.39	0.77	6.85	13.70
33	KPP-1	113	19.1	12.32	0.62	86.26	24.65	1.23	172.52	0.30	5.35	10.70	94.79	189.58
34	KPP-2	113	19.1	12.32	0.62	86.26	24.65	1.23	172.52	0.30	5.35	10.70	94.79	189.58
35	KPP-3	114	19.3	12.45	0.62	87.16	24.90	1.25	174.32	0.29	5.40	10.81	95.78	191.56
36	KPP-4	7.01	1.19	0.77	0.04	5.37	1.54	0.08	10.75	4.74	0.33	0.67	5.91	11.81
37	KPP-5	19.2	3.26	2.10	0.11	14.72	4.21	0.21	29.45	1.73	0.91	1.83	16.18	32.36
38	Nes-1	6.15	1.05	0.68	0.03	4.74	1.35	0.07	9.48	5.37	0.29	0.59	5.21	10.42
39	KPon-1	10.9	1.85	1.19	0.06	8.35	2.39	0.12	16.71	3.05	0.52	1.04	9.18	18.36
40	KPon-2	5.03	0.86	0.55	0.03	3.88	1.11	0.06	7.77	6.56	0.24	0.48	4.27	8.54

Note:
Amount PTWI= Amount (kg) of samples that would need to be consumed per week by a 62-kg adult to reach the EFSA [2]'s recalculated PTWI limit (5642 μg/week) for nickel.
Intake*= Mean weekly intake (MWI; mg/week) of nickel for average level fish (0.70 kg) and molluscs (0.28 kg) consumers (mg/week) and the percentages of EFSA [2]'s recalculated PTWI limit (5642 μg/week based on a 62-kg adult) of nickel for average level (PTWI%*) consumers.
Intake**= Mean weekly intake (MWI; mg/week) of nickel for high-level fish (1.40 kg) and molluscs (0.56 kg) consumers (mg/week) and the percentages of EFSA [2]'s recalculated PTWI limit (5642 μg/week based on a 62-kg adult) of nickel for high level (PTWI%**) consumers.

Table S8. Mean nickel concentrations (mg/kg dry weight (DW) and wet weight (WW)) in various species of marine fishes reported in the literature, including the results of the present study (8 species).

No	Species	Location	DW	WW	References
1.	*Decapterus macrosoma*	Setiu	0.65	0.13	This study
	(CF= 0.20)	Gulf of Aqaba, Red Sea, Jordan	1.09	0.22*	Khalaf *et al.* [3]
2	*Megalaspis cordyla*	Setiu	0.48	0.11	This study
	(CF= 0.23)	Karachi Fish Harbor of Pakistan	-	0.25*	Ahmed and Bat [4]
3	*Otolithes ruber*	Setiu	1.16	0.28	This study
	(CF= 0.24)	Kharg Island, Persian Gulf		0.34*	Abadi *et al.* [5]
		Chabahar Bay, Makoran, Iran	0.11	0.03	Agah [6]
		The northern part of the Hormuz strait (Persian Gulf)	12.5	2.99	Janadeleh and Jahangiri [7]
		North of Persian Gulf	3.18	0.76	Niri *et al.* [8]
		Khuzestan shore, northwest of the Persian Gulf.	42.0	10.1	Hosseini *et al.* [9]
4	*Johnius belangerii*	Setiu	1.14	0.26	This study
	0.23	Musa estuary, Iran	1.96	0.45	Abdollahpur Monikh *et al.* [10]
		Daya Bay's Fishery Resource Reserve, South China Sea	-	0.14*	Gu *et al.* [11]
		Indonesia	-	0.01*	Soegianton and Irawan [11]
		Musa Estuary, Persian Gulf	6.47	1.49	Ravanbakhsh *et al.* [13]
5	*Pampus chinensis*	Setiu	1.71	0.32	This study
	0.19	Cox's Bazar, Bangladesh	0.24	0.05	Rakib *et al.* [14]
6	*Anodontostama chacunda*	Setiu	1.93	0.44	This study
	0.23	Arabian Sea coasts of Pakistan	0.15	0.03	Ahmed *et al.* [15]
7	*Rastrelliger kanagurta*	Setiu	0.64	0.17	This study
	0.26	Coastal waters off Kochi, India	10.9	2.83	Rejomon *et al.* [16]
		Kunduchi fish market in Dar es Salaam, Tanzania	0.14	0.04	Mziray and Kimirei [17]
8	*Scomberomorus commerson*	Setiu	1.52	0.35	This study
	0.23	Coast of Karachi, Pakistan	0.62	0.14	Ahmed *et al.* [18]

		Zhongsha (Macclesfield) Fishing Ground, South China Sea	-	32.3*	Gu *et al.* [19]
9	*Carangoides malabaricus* (CF= 24)	Setiu	1.52	0.36	This study
10	*Atule mate* (CF= 23)	Setiu	0.64	0.15	This study
11	*Selaroides leptolepis* (CF= 24)	Setiu	0.64	0.15	This study
12	*Dendrophysa russelli* (CF= 23)	Setiu	1.56	0.36	This study
13	*Chirocentrus dorab* (CF= 23)	Setiu	1.44	0.33	This study
14	*Himantura walga* (CF= 21)	Setiu	0.65	0.14	This study
15	*Nemipterus hexodon* (CF= 0.20)	Setiu	1.93	0.39	This study
16	*Alectis indica* (CF= 0.21)	Setiu	2.27	0.48	This study
17	*Lactarius lactarius* (CF= 0.21)	Setiu	1.52	0.32	This study
18	*Trichiurus lepturus* (CF= 0.22)	Setiu	2.29	0.50	This study
19	*Arius maculatus* (CF= 0.23)	Setiu	3.84	0.88	This study

Note: The data cited from the literature were recalculated for EDI and THQ based on fish consumption rate (100 g/person/day) and body weight of 62 kg for the adult Malaysian population, according to Nurul Izzah et al. (2016). The data which were originally reported on a dry weight basis were all converted into a wet weight basis based on the conversion factor for each species from this study.

* data that were originally reported on a wet weight basis. ** The maximum value was selected for recalculation.

Table S9. Nickel values of estimated daily intake (EDI), target hazard quotient (THQ), and estimated weekly intake (EWI) calculated based on the present study and cited Ni data in the marine fishes from the literature.

No	Species	Location	Ni EDI	Ni THQ	Ni EWI	References
1.	*Decapterus macrosoma*	Setiu	0.21	0.0105	1.47	This study
	(CF= 0.20)	Gulf of Aqaba, Red Sea, Jordan	0.35	0.0176	2.46	Khalaf *et al.* [3]
2	*Megalaspis cordyla*	Setiu	0.18	0.0089	1.25	This study
	(CF= 0.23)	Karachi Fish Harbor of Pakistan	0.40	0.0202	2.82	Ahmed and Bat [4]
3	*Otolithes ruber*	Setiu	0.45	0.0225	3.14	This study
	(CF= 0.24)	Kharg Island, Persian Gulf	0.55	0.0274	3.84	Abadi *et al.* [5]
		Chabahar Bay, Makoran, Iran	0.04	0.0021	0.30	Agah [6]
		The northern part of the Hormuz strait (Persian Gulf), (2015)	4.83	0.2414	33.79	Janadeleh and Jahangiri [7]
		North of Persian Gulf	1.23	0.0615	8.62	Niri *et al.* [8]
		Khuzestan shore, northwest of the Persian Gulf.	16.26	0.8129	113.81	Hosseini *et al.* [9]
4	*Johnius belangerii*	Setiu	0.42	0.0211	2.96	This study
	0.23	Musa estuary, Iran	0.73	0.0364	5.09	Abdollahpur Monikh *et al.* [10]
		Daya Bay's Fishery Resource Reserve, South China Sea	0.23	0.0113	1.58	Gu *et al.* [11]
		Indonesia	0.01	0.0006	0.08	Soegianton and Irawan [11]
		Musa Estuary, Persian Gulf	2.40	0.1200	16.80	Ravanbakhsh *et al.* [13]
5	*Pampus chinensis*	Setiu	0.52	0.0262	3.67	This study
	0.19	Cox's Bazar, Bangladesh	0.07	0.0037	0.51	Rakib *et al.* [14]
6	*Anodontostama chacunda*	Setiu	0.72	0.0358	5.01	This study
	0.23	Arabian Sea coasts of Pakistan	0.06	0.0028	0.39	Ahmed *et al.* [15]
7	*Rastrelliger kanagurta*	Setiu	0.27	0.0134	1.88	This study
	0.26	Coastal waters off Kochi, India	4.56	0.2279	31.91	Rejomon *et al.* [16]
		Kunduchi fish market in Dar es Salaam, Tanzania	0.06	0.0029	0.41	Mziray and Kimirei [17]
8	*Scomberomorus commerson*	Setiu	0.56	0.0282	3.95	This study

	0.23	Coast of Karachi, Pakistan	0.23	0.0115	1.61	Ahmed *et al.* [18]
		Zhongsha (Macclesfield) Fishing Ground, South China Sea	52.05	2.6024	364.34	Gu *et al.* [19]
9	*Carangoides malabaricus* (CF= 24)	Setiu	0.59	0.0294	4.12	This study
10	*Atule mate* (CF= 23)	Setiu	0.24	0.0119	1.66	This study
11	*Selaroides leptolepis* (CF= 24)	Setiu	0.25	0.0124	1.73	This study
12	*Dendrophysa russelli* (CF= 23)	Setiu	0.58	0.0289	4.05	This study
13	*Chirocentrus dorab* (CF= 23)	Setiu	0.53	0.0267	3.74	This study
14	*Himantura walga* (CF= 21)	Setiu	0.22	0.0110	1.54	This study
15	*Nemipterus hexodon* (CF= 0.20)	Setiu	0.62	0.0311	4.36	This study
16	*Alectis indica* (CF= 0.21)	Setiu	0.77	0.0384	5.38	This study
17	*Lactarius lactarius* (CF= 0.21)	Setiu	0.51	0.0257	3.60	This study
18	*Trichiurus lepturus* (CF= 0.22)	Setiu	0.81	0.0406	5.69	This study
19	*Arius maculatus* (CF= 0.23)	Setiu	1.42	0.0712	9.97	This study

Note: the EFSA [2] recalculated PTWI limit of 5642 μg/week for nickel.

Table S10. Overall statistics of nickel concentrations (mg/kg wet weight) (WW) with recalculation of estimated daily intake (EDI), target hazard quotient (THQ), and estimated weekly intake (EWI) in the 8 marine fish species cited from the literature (17 reports of 17 papers) (N= 17).

	WW	EDI	THQ	EWI
Minimum	0.01	0.01	0.0006	0.08
Maximum	32.27	52.05	2.6024	364.34
Mean	3.07	4.94	0.2472	34.61
Median	0.25	0.40	0.0202	2.82
SD	7.92	12.77	0.6386	89.41
Std Error	1.92	3.10	0.1549	21.69
Skewness	3.24	3.24	3.2383	3.24
Kurtosis	9.34	9.34	9.3372	9.34

Note: Eight marine fish species included were *Decapterus macrosoma*, *Megalaspis cordyla*, *Otolithes ruber*, *Johnius belangerii*, *Pampus chinensis*, *Anodontostama chacunda*, *Rastrelliger kanagurta*, and *Scomberomorus commerson*.

References

1. Yap, C.K.; Cheng, W.H.; Karami, A.; Ismail, A. Health Risk Assessments of Heavy Metal Exposure via Consumption of Marine Mussels Collected from Anthropogenic Sites. *Sci. Total Environ.* **2016**, *553*, 285–296, doi:10.1016/j.scitotenv.2016.02.092.
2. EFSA Panel on Contaminants in the Food Chain (CONTAM); Schrenk, D.; Bignami, M.; Bodin, L.; Chipman, J.K.; del Mazo, J.; Grasl-Kraupp, B.; Hogstrand, C.; Hoogenboom, L. (Ron); Leblanc, J.; et al. Update of the Risk Assessment of Nickel in Food and Drinking Water. *EFSA J.* **2020**, *18*, doi:10.2903/j.efsa.2020.6268.
3. Khalaf, M.; Al-Najjar, T.; Alawi, Dr.M.; A.Disi, A. Levels of Trace Metals in Three Fish Species Decapterus Macrellus, Decapterus Macrosomos and Decapterus Russelli of the Family Carangidae from the Gulf of Aqaba, Read Sea, Jordan. *Nat. Sci.* **2012**, *4*, 362–367, doi:10.4236/ns.2012.46050.
4. Ahmed, Q.; Bat, L. Potential Risk of Some Heavy Metals in Pampus Chinensis (Euphrasen) Chinese Silver Pomfret Stromateidae Collected from Karachi Fish Harbour, Pakistan. *Int. J. Mar. Sci.* **2015**, *555*, 1–5, doi:10.5376/ijms.2015.05.0021.
5. Abadi, D.R.V.; Dobaradaran, S.; Nabipour, I.; Lamani, X.; Ravanipour, M.; Tahmasebi, R.; Nazmara, S. Comparative Investigation of Heavy Metal, Trace, and Macro Element Contents in Commercially Valuable Fish Species Harvested off from the Persian Gulf. *Environ. Sci. Pollut. Res. Int.* **2015**, *22*, 6670–6678, doi:10.1007/s11356-014-3852-1.
6. Agah, H. Ecological Risk Assessment of Heavy Metals in Sediment, Fish, and Human Hair from Chabahar Bay, Makoran, Iran. *Mar. Pollut. Bull.* **2021**, *169*, 112345, doi:10.1016/j.marpolbul.2021.112345.
7. Janadeleh, H.; Jahangiri, S. Risk Assessment and Heavy Metal Contamination in Fish (Otolithes Ruber) and Sediments in Persian Gulf. *J. Community Health Res.* **2016**, *5*, 169–181.
8. Niri, A.S.; Sharifian, S.; Ahmadi, R. Assessment of Metal Accumulation in Two Fish Species (Tenualosa Ilisha and Otolithes Ruber), Captured from the North of Persian Gulf. *Bull. Environ. Contam. Toxicol.* **2015**, *94*, 71–76, doi:10.1007/s00128-014-1429-9.
9. Hosseini, M.; Nabavi, S.M.B.; Nabavi, S.N.; Pour, N.A. Heavy Metals (Cd, Co, Cu, Ni, Pb, Fe, and Hg) Content in Four Fish Commonly Consumed in Iran: Risk Assessment for the Consumers. *Environ. Monit. Assess.* **2015**, *187*, 237, doi:10.1007/s10661-015-4464-z.
10. Abdolahpur Monikh, F.; Safahieh, A.; Savari, A.; Ronagh, M.T.; Doraghi, A. The Relationship between Heavy Metal (Cd, Co, Cu, Ni and Pb) Levels and the Size of Benthic, Benthopelagic and Pelagic Fish Species, Persian Gulf. *Bull. Environ. Contam. Toxicol.* **2013**, *90*, 691–696, doi:10.1007/s00128-013-0986-7.
11. Gu, Y.-G.; Huang, H.-H.; Lin, Q. Concentrations and Human Health Implications of Heavy Metals in Wild Aquatic Organisms Captured from the Core Area of Daya Bay's Fishery Resource Reserve, South China Sea. *Environ. Toxicol. Pharmacol.* **2016**, *45*, 90–94, doi:10.1016/j.etap.2016.05.022.

12. Soegianto, A.; Irawan, B.; Hamami Bioaccumulation of Heavy Metals in Aquatic Animals Collected from Coastal Waters of Gresik, Indonesia. In *Coastal Environments: Focus on Asian Regions*; Subramanian, V., Ed.; Springer Netherlands: Dordrecht, 2012; pp. 144–154 ISBN 978-90-481-3001-6.
13. Ravanbakhsh, M.; Zare Javid, A.; Hadi, M.; Jaafarzadeh Haghighi Fard, N. Heavy Metals Risk Assessment in Fish Species (Johnius Belangerii (C) and Cynoglossus Arel) in Musa Estuary, Persian Gulf. *Environ. Res.* **2020**, *188*, 109560, doi:10.1016/j.envres.2020.109560.
14. Rakib, M.R.J.; Jolly, Y.N.; Enyoh, C.E.; Khandaker, M.U.; Hossain, M.B.; Akther, S.; Alsubaie, A.; Almalki, A.S.A.; Bradley, D.A. Levels and Health Risk Assessment of Heavy Metals in Dried Fish Consumed in Bangladesh. *Sci. Rep.* **2021**, *11*, 14642, doi:10.1038/s41598-021-93989-w.
15. Ahmed, Q.; Bat, L.; Mohammad, Q. Bioaccumulation of Nine Heavy Metals in Some Tissues of Anodontostoma Chacunda (Hamilton, 1822) in the Arabian Sea Coasts of Pakistan. *Nat. Eng. Sci.* **2017**, *2*, 79–92, doi:10.28978/nesciences.349296.
16. Rejomon, G.; Nair, M.; Joseph, T. Trace Metal Dynamics in Fishes from the Southwest Coast of India. *Environ. Monit. Assess.* **2010**, *167*, 243–255, doi:10.1007/s10661-009-1046-y.
17. Mziray, P.; Kimirei, I. Bioaccumulation of Heavy Metals in Marine Fishes (Siganus Sutor, Lethrinus Harak, and Rastrelliger Kanagurta) from Dar Es Salaam Tanzania. *Reg. Stud. Mar. Sci.* **2016**, *7*, 72–80, doi:10.1016/j.rsma.2016.05.014.
18. Ahmed, Q.; Bat, L.; Yousuf, F.; Mohammad Ali, Q.; Nazim, K. Accumulation of Heavy Metals (Fe, Mn, Cu, Zn, Ni, Pb, Cd and Cr) in Tissues of Narrow-Barred Spanish Mackerel (Family-Scombridae) Fish Marketed by Karachi Fish Harbor. *Open Biol. Sci. J.* **2015**, *1*, 20–28, doi:10.2174/2352633501501010020.
19. Gu, Y.-G.; Lin, Q.; Wang, X.-H.; Du, F.-Y.; Yu, Z.-L.; Huang, H.-H. Heavy Metal Concentrations in Wild Fishes Captured from the South China Sea and Associated Health Risks. *Mar. Pollut. Bull.* **2015**, *96*, 508–512, doi:10.1016/j.marpolbul.2015.04.022.
